# Heating the Cold: Overcoming Immunotherapy Resistance in Microsatellite-Stable Colorectal Cancer: A Systematic Review

**DOI:** 10.3390/molecules31173124

**Published:** 2026-09-06

**Authors:** Dorota Bartusik-Aebisher, Daniel Roshan Justin Raj, Izabella Wilk, David Aebisher

**Affiliations:** 1 Department of Biochemistry and General Chemistry, Faculty of Medicine, Collegium Medicum, University of Rzeszow, 35-310 Rzeszow, Poland; dbartusikaebisher@ur.edu.pl; 2 English Division Science Club, Faculty of Medicine, Collegium Medicum, University of Rzeszow, 35-310 Rzeszow, Poland; dj135639@stud.ur.edu.pl (D.R.J.R.); iw135750@stud.ur.edu.pl (I.W.); 3 Department of Photomedicine and Physical Chemistry, Faculty of Medicine, Collegium Medicum, University of Rzeszow, 35-310 Rzeszow, Poland

**Keywords:** colorectal cancer, microsatellite-stable colorectal cancer, immunotherapy resistance, immune checkpoint inhibitors, cold tumours, hot tumours, tumour microenvironment, immune exclusion, combination therapy, tumour immune reprogramming

## Abstract

Colorectal cancer (CRC) has shown significant heterogeneity regarding its response to immunotherapy. Long-lasting, beneficial effects have been observed in mismatch repair-deficient, microsatellite instability-high (dMMR/MSI-H) tumours, while mismatch repair-proficient, microsatellite stable (pMMR/MSS) tumours have remained resistant. Such differences in results have been studied in this review through the “hot” and “cold” tumour concept. It explains how various biological and microenvironmental factors play a role in immune resistance and T-cell priming and infiltration. Key factors include a low neoantigen load and defects in antigen presentation, which reduce the overall immune recognition of tumour cells. The review also studies certain processes such as Wnt/β-catenin and mitogen-activated protein kinase (MAPK) signalling and what input they have in the prevention of effective antitumour immune responses. Conventional treatments like chemotherapy and radiotherapy have been considered alongside more targeted treatments such as the inhibition of vascular endothelial growth factor (VEGF) signalling and the suppression of myeloid-mediated immune evasion, to convert “cold” MSS tumours into immune-responsive lesions. Methods which aim to modify the tumour microenvironment such as metabolic reprogramming and microbiome modulation have also been covered in this review. Artificial intelligence and nanomedicine are new technologies that could provide improved patient stratification and therapeutic precision, although their clinical application in pMMR/MSS CRC remains under investigation. A systematic literature search of PubMed and PubMed Central (PMC) was conducted from 10 June 2026 to 19 August 2026 using predefined eligibility criteria, with study selection reported according to PRISMA 2020. Because of substantial heterogeneity in study design, therapeutic approach and reported outcomes, the included evidence was synthesized narratively rather than by meta-analysis. A total of 158 studies were included.

## 1. Introduction

Colorectal cancer (CRC) is one of the most commonly diagnosed cancers worldwide. In many countries, it remains a massive health burden as there have been more than an estimated 1.9 million new cases and 930,000 deaths just in 2020 [1]. Although there have been numerous developments recently in screening, surgery, chemotherapy and molecularly targeted treatment, the results still remain poor for advanced and metastatic CRC. This has raised more interest in finding new systemic strategies to tackle the disease [2]. One strategy that has reshaped modern oncology is immune checkpoint inhibitors (ICIs). They have the ability to restore the responses of exhausted antitumour T-cells by blocking some of the key inhibitory pathways. These include the programmed cell death protein 1 (PD-1), programmed cell death-ligand 1 (PD-L1), and cytotoxic T-lymphocyte associated antigen 4 (CTLA-4) [3]. Such a therapeutic advancement is very important in CRC. It has shown that immunologic context more strongly decides the benefits from immunotherapy rather than just the location of the tumour [4]. This concept can be illustrated clearly by the differences between mismatch repair-deficient, microsatellite instability-high (dMMR/MSI-H) tumours and mismatch repair-proficient, microsatellite stable (pMMR/MSS) tumours. dMMR/MSI-H CRCs have a large number of mutations and generate many neoantigens. They often show dense T-cell infiltration and a higher expression of immune checkpoints, which results in an inflamed tumour microenvironment that is susceptible to a PD-1 blockade [5]. KEYNOTE-177 and CheckMate-142 are remarkable clinical studies that have used this biology. These studies saw that pembrolizumab- or nivolumab-based regimens had given durable responses and valuable survival benefits in dMMR/MSI-H metastatic CRC [6,7,8]. On other hand, pMMR/MSS tumours, which make up roughly 85% of all CRCs and the much greater majority of metastatic cases, responded rather poorly to the checkpoint blockade [5,9]. These tumours usually have a lower neoantigen burden, a weaker T-cell infiltration, immune exclusion, and a tumour microenvironment that is much more suppressive. This forms the central paradox in CRC immunotherapy, which is that the patients who are in need of better treatment are oftentimes the ones who benefit the least from current ICIs [10].

“Hot” and “cold” tumours represent two very contrasting immune states within the tumour microenvironment. Hot tumours are typically T-cell-inflamed and are enriched in interferon-γ-related signalling. They are also marked by a large number of intratumoral clusters of differentiation 8-positive (CD8+) T-cells, a higher neoantigen load, and an increased expression of immune checkpoints such as PD-1 and PD-L1. These are features that make them much more likely to respond to the inhibition of immune checkpoints [11,12,13]. In comparison, cold tumours are not well infiltrated by effector T-cells and show immune cells being restricted within the tumour margin rather than being able to enter the tumour core. This indicates immune exclusion and not antitumour activity that is productive [14,15]. A lower antigenicity alongside stromal, vascular, and myeloid barriers are seen in these tumours, which inhibit T-cell priming, trafficking, or functioning [16,17]. This difference is particularly important in colorectal cancer because most pMMR/MSS tumours display a phenotype that is cold or immune-excluded, helping to explain why they have such low sensitivity to immunotherapy [18,19]. These biological features become huge clinical hurdles for immune checkpoint inhibitors in MSS CRC. Even though the PD-1 blockade had shown solid benefits in dMMR/MSI-H disease, the response rates in MSS CRC still remained very low across most trials of single agent PD-1 or PD-L1 inhibition [6,20]. Similarly, PD-1/CTLA-4 based strategies also have not been able to provide useful activity when they are used in unselected MSS tumours as monotherapy or near-monotherapy [21,22]. Such a failure can be explained by the fact that although the checkpoint blockade can release the existing antitumour T-cell responses, it cannot produce a strong enough immune response. This is especially true in the tumours which have T-cells which are scarce, excluded, or functionally suppressed [23,24]. Inconsistent and minimal benefits were observed even with combination regimens in MSS CRC. This highlights that the biggest challenge is to convert these immunologically cold lesions into tumours that are inflamed and T-cell-accessible so that ICIs can respond to them [25]. The current European Society for Medical Oncology (ESMO) guidelines reflect this difference. dMMR/MSI-H status is an important factor in selecting patients for immunotherapy while pMMR/MSS metastatic CRC is generally treated according to molecular characteristics and established systemic and targeted therapies [26].

This review will focus on why most of the MSS colorectal cancers are resistant to immunotherapy, even with the success of checkpoint blockade in MSI-H disease. It studies how tumours are made to be immunologically “cold” by factors such as poor antigenicity, ineffective T-cell recruitment, and a tumour microenvironment that is immunosuppressive [2,16]. It then considers treatment combinations that can possibly reverse this resistance by more than simply adding more drugs. Some of these treatments include chemotherapy, radiotherapy, and targeted therapy, which all can improve immune reactions in the tumour and provide better sensitivity to immune checkpoint inhibitors [27]. An important theme that will be carried throughout is the idea of “heating” these “cold” tumours by shifting them into a state where they are more inflamed and T-cell-accessible. These features will be essential for the tumours to respond to immunotherapy [11]. As such, this paper will connect the mechanisms of resistance with various translational strategies that could increase the benefits of immunotherapy beyond the small MSI-H responsive subgroup. Accordingly, this systematic review aimed to identify and synthesize the biological mechanisms underlying immunotherapy resistance in pMMR/MSS CRC and to evaluate the preclinical, translational and clinical evidence for therapeutic strategies designed to overcome these mechanisms.

## 2. Materials and Methods

### 2.1. Review Design and PRISMA Compliance

This systematic review was conducted using predefined eligibility, screening and data-extraction procedures and is reported in accordance with the Preferred Reporting Items for Systematic Reviews and Meta-Analyses (PRISMA) 2020 statement. Owing to substantial methodological, clinical and outcome heterogeneity among the included studies, a narrative synthesis was performed rather than a meta-analysis. The completed PRISMA 2020 checklist is provided as Supplementary Table S1. This systematic review was not prospectively registered, and a separate review protocol was not prepared.

### 2.2. Eligibility Criteria

English-language, full-text journal articles relevant to immunotherapy resistance and therapeutic sensitization were considered. The evidence base included human clinical studies, translational investigations, in vivo experimental studies, and in vitro mechanistic studies relevant to pMMR/MSS colorectal cancer and the biological mechanisms underlying immune resistance. Primary clinical studies included randomized and non-randomized trials, early-phase and single-arm studies, and other prospective or retrospective investigations where relevant. Studies conducted in other tumour types were considered only when they provided mechanistic or translational evidence directly applicable to the resistance pathways or therapeutic strategies discussed in pMMR/MSS colorectal cancer.

Systematic reviews, meta-analyses, narrative reviews, and clinical guidelines were used to provide background, contextual interpretation, and supporting evidence, but were distinguished from primary studies when evaluating therapeutic efficacy. Conference abstracts and case reports were not included because of their limited methodological and outcome information. The final reference set contained publications from 2010 to 2026.

### 2.3. Information Sources and Search Strategy

PubMed and PubMed Central (PMC) were systematically searched from 10 June 2026 to 19 August 2026. The final search was conducted on 19 August 2026. Search terms combined concepts relating to colorectal cancer, mismatch repair proficiency/microsatellite stability, immunotherapy, immune checkpoint inhibition and treatment resistance. The complete search strategy, including Boolean operators and any filters or limits applied, is provided in Supplementary Table S2.

### 2.4. Study Selection

Following duplicate removal, two reviewers independently screened the titles and abstracts of retrieved records against the predefined eligibility criteria. Potentially eligible reports subsequently underwent full-text assessment by the same reviewers. Disagreements regarding eligibility were resolved by discussion and, where necessary, consultation with a third reviewer.

### 2.5. Data Extraction and Data Items

Data were extracted from eligible reports using a standardized data-extraction framework. The information collected included publication year, study design, experimental or clinical population, sample size, pMMR/MSS status, intervention and comparator where applicable, therapeutic target or resistance mechanism, study phase, treatment outcomes, relevant immunological or mechanistic findings, and major reported limitations. Methodological quality, risk of bias, reporting bias and certainty of evidence were not formally assessed, and this is considered as a limitation of this review. For clinical studies, effect measures were extracted as reported in the original publications, including hazard ratios with 95% confidence intervals for time-to-event outcomes and response proportions where available. Because the findings were synthesized narratively, effect estimates were not statistically pooled. All data supporting this systematic review were obtained from the cited publications and are summarized within the article and Supplementary Table S4. No separate analytic code or unpublished dataset was generated for this review.

## 3. Results

**Figure 1:**
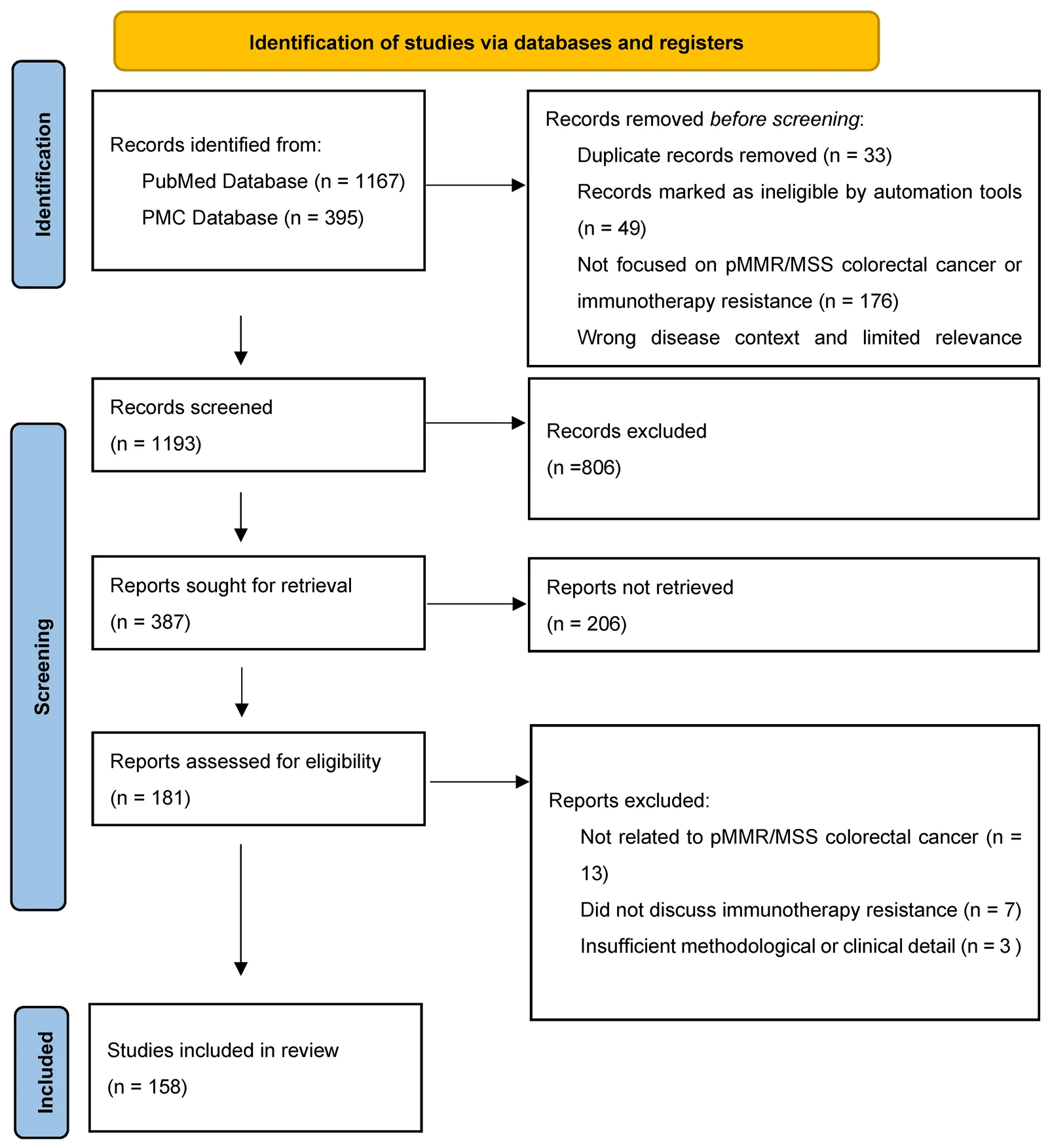
Preferred Reporting Items for Systematic Reviews and Meta-Analyses (PRISMA) flow diagram that shows the identification, screening, eligibility assessment, and inclusion of articles for this review. A total of 158 studies were selected and included in the final review.

The search identified 1562 records, of which 1193 were screened. A total of 387 reports were sought for retrieval, of which 206 could not be retrieved and 181 underwent full-text eligibility assessment. Following the exclusion of 23 reports, 158 studies were included in the review (Figure 1). Full-text exclusions and their reasons are provided in Supplementary Table S3, while the characteristics of included studies are summarized in Supplementary Table S4.

The included evidence ranged from preclinical and translational studies to early-phase and randomized clinical trials. Methodological limitations were more pronounced in small, single-arm and early-phase studies, particularly because of limited sample sizes and the absence of randomized comparators, whereas randomized phase II and III trials generally provided more robust evidence.

Representative clinical outcomes and effect estimates are reported in Figure 1. Overall, randomized studies generally showed negative or modest clinical benefit in unselected pMMR/MSS CRC, whereas more encouraging signals were mainly observed in small, early-phase or biomarker-selected studies.

Because of substantial heterogeneity in the study design, populations, interventions and outcomes, findings were synthesized narratively and no meta-analysis, statistical heterogeneity analysis or sensitivity analysis was performed. Publication bias was not assessed statistically. Certainty was interpreted according to study design, risk of bias and developmental stage, rather than formal GRADE assessment, with randomized evidence given greater weight than early-phase, translational or preclinical findings.

## 4. Mechanistic Hierarchy of Immunotherapy Resistance in pMMR/MSS CRC

Immunotherapy resistance in pMMR/MSS CRC develops due to several related levels of immune escape. They can be broadly classified into tumour-intrinsic mechanisms, stromal and vascular immune exclusion, metabolic suppression, and microbiome-associated effects. These mechanisms can cause either primary resistance or acquired resistance after an initial therapeutic response.

### 4.1. Tumour-Intrinsic Mechanisms of Resistance

Tumour-intrinsic features play an important role in keeping many mismatch repair-proficient colorectal cancers immunologically “cold”. The potential of generating immunogenic neoantigens is significantly reduced by low tumour mutational burden. In turn, spontaneous T-cell priming could be limited and the responsiveness to immune checkpoint blockade may become weakened [28]. Even if there is a presence of tumour antigens, a defect in antigen presentation could prevent effective immune recognition. The downregulation or loss of the major histocompatibility complex class I, in particular, does not allow the tumour-derived peptides to be presented to CD8+ cytotoxic T-cells. Due to this, the malignant cells would be able to evade immune surveillance, which results in both primary and acquired resistance to immunotherapy [29,30]. At the same time, oncogenic signalling pathways can form an immune-excluded microenvironment. Abnormal Wnt/β-catenin signalling has also been observed to be linked with poor dendritic cell recruitment and defective T-cell infiltration, which lead to a sustained and non-inflamed tumour [31,32]. Immune escape can also be promoted by the activation of the mitogen-activated protein kinase (MAPK) pathway. It can achieve this through its ability to reduce the recruitment of effector cells, alter the cytokine signalling, and support suppressive tumour programmes, which further blocks antitumour immunity [33]. Overall, antigen recognition and T-cell recruitment become affected by these tumour-intrinsic mechanisms, which gives a biological rationale for therapeutic strategies aiming to restore tumour immunogenicity and immune priming.

### 4.2. Immunosuppressive Cellular Microenvironment

Tumour microenvironment-mediated immunosuppression is one of the biggest contributors of checkpoint-blockade resistance. The antitumour immunity can be suppressed by regulatory T-cells through the release of interleukin-10 (IL-10) and transforming growth factor-beta (TGF-β), and also by the contact-dependent inhibition of effector T-cells. Through this suppression, local immune tolerance can be maintained correctly, and cytotoxic activity will be reduced [34]. Key amino acids can be depleted by myeloid-derived suppressor cells (MDSCs), which leads to the production of reactive oxygen and nitrogen species, defective priming of the T-cells, and strengthening the expansion of T-regulatory cells [35]. Similarly, by ways of secreting IL-10 and vascular endothelial growth factor (VEGF), immune escape is reinforced by tumour-associated macrophages (TAMs) that have an M2-like phenotype. They also release matrix-remodelling mediators that can support angiogenesis, tissue repair programmes, and T-cell dysfunction rather than the clearance of the tumour [36,37]. All of these tumour microenvironment components form an immunosuppressive and T-cell excluded state, leading to ineffective antigen-specific killing and resistance that is still withheld even when the checkpoint pathways have been therapeutically blocked [38].

### 4.3. Stromal and Vascular Immune Exclusion

A distinct stromal barrier is made by the cancer-associated fibroblasts as they remodel the extracellular matrix and produce chemokines such as C-X-C motif chemokine ligand 12 (CXCL12). This barrier will create a physical and biochemical environment that separates CD8+ T cells from tumour nests [39,40]. Checkpoint blockade is often unsuccessful because of the challenges posed by immune exclusion as the cytotoxic T-cells have been found to accumulate at the invasive margin, but not be able to enter the tumour core. This is caused mainly by dense extracellular matrix remodelling, in which a stiff and highly organized physical barrier is formed. It directly limits the movement of lymphocytes through the tumour bed [41,42]. Cancer-associated fibroblasts play a role in this process by depositing additional collagen and other matrix components, which further strengthens the barrier. Stromal tracks that support the progression of tumours and restrict access for immune cells are also formed [43]. Consequently, even when there are antitumour T-cells being generated, their distance and separation from the malignant cells may become detrimental to the efficiency of immunotherapy. Abnormal vasculature forms a complementary barrier. In angiogenesis that is driven by VEGF, it can be observed that the vessels are not well organized, with low perfusion, high permeability, and an abnormal endothelial function. These are all features that obstruct the trafficking of effector T-cells into the tumour tissue [44]. Such vascular dysfunction can also give rise to conditions like hypoxia and acidosis, which support immune suppression and maintain a non-inflamed microenvironment. Additionally, direct immunomodulatory effects are caused by VEGF as it limits the recruitment of T-cells and helps the stromal and myeloid programmes that are suppressive in nature [45]. Matrix remodelling and vascular abnormalities together form a structural and functional barrier against immune invasion. These structural barriers form the mechanical basis for stromal-targeting and vascular normalization strategies discussed in Section 5.

### 4.4. Metabolic and Hypoxic Immunosuppression

Antitumour immunity is actively held back by the biochemical microenvironment that the tumour metabolism creates. When aerobic glycolysis fuels the accumulation of lactate, the tumour environment is acidified, leading to the impairment of T-cell proliferation, cytokine production, and cytotoxic activity. The impairment occurring across several key components causes the suppressive immune populations to persist even longer [46]. Indoleamine 2,3-dioxygenase (IDO)-mediated tryptophan catabolism is another major mechanism that is responsible for using up amino acids that are required for T-cell effector functions. It also promotes T-cell anergy, apoptosis, and regulatory phenotypes [47]. Hypoxia is a condition that is known to further intensify this suppressive state. It does this by stabilizing the hypoxia-inducible signalling, reducing the trafficking and function of immune cells. These obstructions stimulate the production of inhibitory metabolites and cytokines inside the tumour microenvironment [48,49]. The excess of lactate, depletion of tryptophan, and hypoxia provide a metabolically hostile and ideal environment to maintain immune exclusion and continue being resistant to the checkpoint blockade [50]. Thus, metabolic reprogramming can complement the checkpoint blockade by restoring a microenvironment that is more permissive to effector T-cell function.

### 4.5. Microbiome-Associated Immunotherapy Resistance

The gut microbiome can also influence responsiveness to immune checkpoint blockade. Gut dysbiosis is frequently associated with weaker anti-PD-1 activity in many clinical studies. Quite the opposite can be observed when there are richer and more favourable communities, correlating with much better systemic and intratumoral immunity [51,52]. Dysbiosis involving the bacteria *Fusobacterium nucleatum* is repeatedly linked with colorectal cancer and has shown to be involved with an immunosuppressive microenvironment. Patients that were diagnosed with metastatic CRC and had experienced failure with immunotherapy expressed an abundance of *F. nucleatum* and increased levels of succinic acid. Such findings support the narrative that it plays a functional role in resistance, rather than being just a passive association [53]. Reviews that are based on the checkpoint blockade further support the claim that the disruption of the microbiome may impair antigen presentation, the priming of T-cells, and effector function, thereby elongating immune exclusion [54]. Any exposure to antibiotics can further exacerbate this problem as they eliminate beneficial commensals during the peri-immunotherapy period. The use of antibiotics has also been connected with poorer results in patients who took immune checkpoint inhibitors, as suggested by clinical and meta-analytic evidence [55,56].

### 4.6. Temporal Patterns of Resistance: Primary and Acquired Resistance

**Figure 2:**
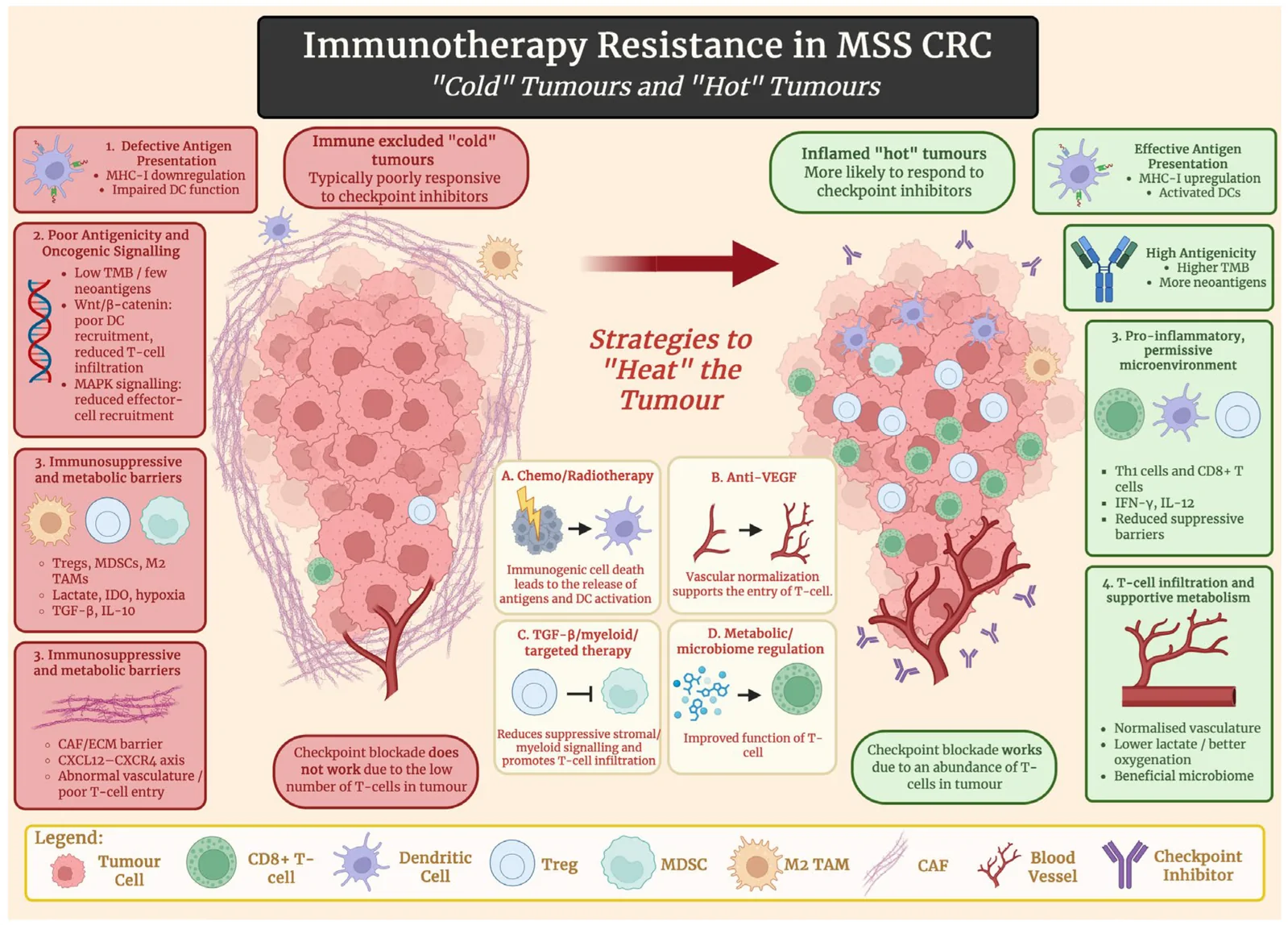
Major mechanisms of immunotherapy resistance in pMMR/MSS colorectal cancer and their contribution to an immunologically “cold” tumour state. Tumour-intrinsic alterations, immunosuppressive cells, stromal and vascular barriers, and metabolic dysfunction can impair antigen presentation, T-cell priming, immune-cell infiltration and effector function, thereby limiting responsiveness to immune checkpoint blockade. Abbreviations from the figure: dendritic cell (DC), major histocompatibility complex class I (MHC-I), tumour mutational burden (TMB), indoleamine 2,3-dioxygenase (IDO), myeloid-derived suppressor cell (MDSC), tumour-associated macrophage (TAM), transforming growth factor-beta (TGF-β), interleukin-10 (IL-10), cancer-associated fibroblast (CAF), extracellular matrix (ECF), C-X-C motif chemokine ligand 12 (CXCL12), C-X-C chemokine receptor 4 (CXCR4), vascular endothelial growth factor (VEGF), and interferon-gamma (IFN-γ). Created in BioRender. Roshan, D. (2026) https://BioRender.com/hkb4zcf.

In addition to the classification according to biological mechanism, immunotherapy resistance can be distinguished temporally as primary or acquired resistance. Primary resistance is when there is a failure to produce a meaningful response to immune checkpoint inhibitors. Acquired resistance, however, only emerges after an early response or the stabilization of disease and is shortly followed by a renewed tumour progression [57]. Primary resistance is usually characterized in “cold” tumours by the absence of T-cell priming, poor antigen presentation, or the exclusion of lymphocytes from the tumour bed [58]. The loss of tumour responsiveness to interferon-gamma (IFN-γ) is a well-known mechanism that prevents the effective checkpoint-based immune activation, associating itself with non-response from the beginning [59]. Likewise, Janus kinase 1/2 (JAK1/2)-inactivating mutations, even in mismatch repair-deficient colon cancer, are related to primary resistance in the PD-1 blockade [60]. Acquired resistance, on the other hand, is the adaptive escape under therapeutic pressure. Here, tumours that were initially inflamed advance by way of immunoediting, losing neoantigens, restricting antigen presentation, and by blocking the interferon-response pathways [61]. This can be seen clinically in tumours that, after developing defects in JAK1/2 or β2-microglobulin, relapsed on the PD-1 blockade, allowing them to escape recognition by T-cells, even though there had been an earlier response [62]. Being able to differentiate between primary and acquired resistance is very important. This is because primary resistance requires the shift of non-inflamed tumours into T-cell-inflamed lesions and acquired resistance calls for strategies that can prevent or reverse adaptive immune escape [63], as seen in Figure 2.

## 5. Mechanism-Based Strategies to Convert “Cold” MSS CRC into “Hot” Tumours

The resistance mechanisms discussed in Section 4 give distinct therapeutic targets for converting immunologically “cold” pMMR/MSS CRC into more inflamed and T-cell-accessible tumours. Heating strategies can therefore be organized according to the barrier that they are designed to overcome. Barriers include insufficient antigen presentation and priming, cellular and stromal immunosuppression, abnormal vasculature, metabolic suppression and resistance due to the microbiome. Additional approaches attempt to directly activate innate immune sensing and pro-inflammatory signalling. The strategies discussed below have different stages of development. Mechanistic findings from primarily preclinical or translational studies are distinguished from preliminary early-phase clinical signals. Efficacy has been clinically considered only when it could be supported by appropriate randomized comparative evidence.

### 5.1. Restoring Antigen Release and T-Cell Priming

Restoring tumour antigenicity and T-cell priming is a major strategy for overcoming immune resistance [64]. Radiotherapy has been shown to stimulate immunogenic cell death, leading to the exposure of calreticulin, release of adenosine triphosphate (ATP), and high-mobility group box 1 (HMGB1) signalling. These factors improve dendritic cell activation, cross-presentation, and subsequent CD8+ T-cell priming [65]. In such circumstances, the irradiated tumours can adopt a function as an in situ vaccine and further enhance antitumour immunity [66]. Oncolytic viruses add on to this effect by selectively killing malignant cells, all while inducing local inflammation, increasing the release of antigens, and recruiting antigen-presenting cells and effector lymphocytes into the previously non-inflamed lesions [67,68]. Personalized neoantigen vaccines are another strategy that expands tumour-specific T-cell clones. They can do this by expanding the tumour-specific T-cell clones and boosting the responses of clusters of differentiation 4-positive (CD4+) and CD8+ T-cells against the targets that are absent from normal tissue [69]. These approaches together try to increase antigen visibility, improve priming in lymphoid organs and tumours, and provide a microenvironment that is more inflamed and better suited to respond to a checkpoint blockade. This is particularly important in mismatch repair-proficient colorectal cancer where the baseline neoantigenicity and spontaneous T-cell infiltration is often low.

### 5.2. Reversing Immunosuppressive Cellular and Stromal Signalling

Regulatory T-cells, MDSCs, and TAMs together have been observed to suppress cytotoxic lymphocytes, antigen presentation, and assist with the exclusionary cytokine networks [70,71]. To reprogram or destroy these cells, new myeloid-directed strategies are being adopted. One such strategy is the colony stimulating factor 1 receptor (CSF1R) blockade which can reduce or reprogram TAMs and bring back antitumour immunity. Other strategies include the C-C chemokine receptor 2 (CCR2) and C-X-C chemokine receptor 2 (CXCR2) which can possibly obstruct the entry of suppressive monocytes and granulocytic MDSCs into the tumours [72]. TGF-β blockade is an attractive method in immune-excluded tumours where it can relax the stromal barrier to stimulate the entry of CD8+ T-cells and even sensitize some cancers that were previously resistant to checkpoint inhibition [73]. These interventions attempt to condition the tumour environment and target the suppressive cellular and stromal barriers described in Section 4.2 and Section 4.3, with the aim of restoring T-cell infiltration and function.

### 5.3. Normalizing Tumour Vasculature and Restoring Immune Trafficking

Building on the vascular barriers described in Section 4.3, VEGF/VEGFR inhibition aims to normalize tumour vasculature, improve oxygenation and facilitate T-cell trafficking [44,74]. VEGF is a direct inhibitor of antitumour immunity as it hinders the maturation of dendritic cells and promotes the expansion of T-regulatory cells and MDSCs. Leukocyte extravasation is also blocked as it limits endothelial adhesion signals [75,76]. Preclinical studies have suggested that VEGF inhibition may lead to an improvement in immune infiltration and enhance tumour control when combined with T-cell based or PD-1 directed immunotherapy. An increase in the accumulation of CD8+ T-cells was also reported [77]. These findings provide a strong mechanistic basis for vascular normalization. Its clinical benefit in pMMR/MSS CRC, however, depends on the treatment regimen and requires further validation in randomized studies.

### 5.4. Reversing Metabolic and Hypoxic Immunosuppression

Metabolic reprogramming can restore effector lymphocyte function by reducing nutrient and pH-related barriers [78]. IDO inhibition is a rational partner for checkpoint blockade. Tryptophan catabolism caused by IDO-1 diminishes tryptophan while accumulating kynurenine, and also promotes the dysfunction and tolerance of T-cells [79,80]. The failure of the ECHO-301 clinical trial provided important lessons regarding the patient selection and combination design [81]. Lactate-targeted strategies such as the inhibition of monocarboxylate transporter 1 (MCT1) and monocarboxylate transporter 4 (MCT4) are also similarly promising. The inhibition can reduce extracellular acidosis, show improvements in leukocyte infiltration, and restore the activity of CD8+ T-cells, which improves the efficacy of anti-PD-1 in preclinical models [82,83]. Limiting the circulation of lactate can weaken immune exclusion and reduce metabolic competition within the tumour microenvironment. This is because lactate also supports myeloid programmes which are suppressive [84]. Glutamine metabolism represents another potential therapeutic target which reduces the anabolic fitness of the tumour. Additionally, it lowers IDO-associated immunosuppression, and increases antitumour immunity, especially when it is paired with immune checkpoint blockade [85]. Approaches like these aim to shift the microenvironment from being tolerogenic and depleted of nutrients to being T-cell-permissive and therapeutically responsive.

### 5.5. Modulating Microbiome-Associated Resistance

Microbiome modulation is an emerging approach for improving immunotherapy responsiveness. Fecal microbiota transplantation (FMT) in patients that were diagnosed with checkpoint-refractory melanoma has brought back sensitivity to anti-PD-1 in some subsets of patients. The restored sensitivity was also accompanied with shifts in microbial composition and tumour immune programmes [86,87]. In the open-label, single-arm phase II RENMIN-215 trial, 20 patients with refractory MSS metastatic CRC received FMT combined with tislelizumab and fruquintinib. The study produced an objective response rate of 20%, with a median progression-free survival of 9.6 months and a median overall survival of 13.7 months [88]. An updated analysis after a median follow-up of 34 months reported sustained antitumour activity with a consistent safety profile [89]. The idea that FMT may enhance responsiveness to PD-1 blockade is further supported by evidence from patients with anti-PD-1-refractory advanced solid tumours, which also include gastrointestinal cancers [90]. However, the independent contribution of FMT could not be established as RENMIN-215 was small and single-arm, and combined FMT with both anti-PD-1 and anti-angiogenic therapy. Therefore, until its benefit is confirmed in larger studies, FMT should remain just as an investigational strategy in MSS CRC. These findings together support the further development of microbiome modulation as a potential means of improving ICI responsiveness, even though its clinical role in pMMR/MSS CRC remains uncertain. Conversely, dysbiosis has been associated with resistance to ICIs [51,52]. Diet is another modifiable factor where a higher intake of fibre was noticed to give better checkpoint blockade results. Most likely, this is because of the support given from metabolites to antigen presentation and T-cell function [91]. Probiotics, however, are a factor that is more uncertain, with some reviews placing importance on the specificity of the strain and the little standardization in routine oncologic usage [92]. Microbiome-assisted priming can be viewed as a supplementary method alongside immunotherapy, as suggested by these data. However, it is important to note that trials involving CRC must specify their donors, diets, formulations and safety levels.

### 5.6. Activating Innate Immune Sensing and Cytokine Signalling

**Figure 3:**
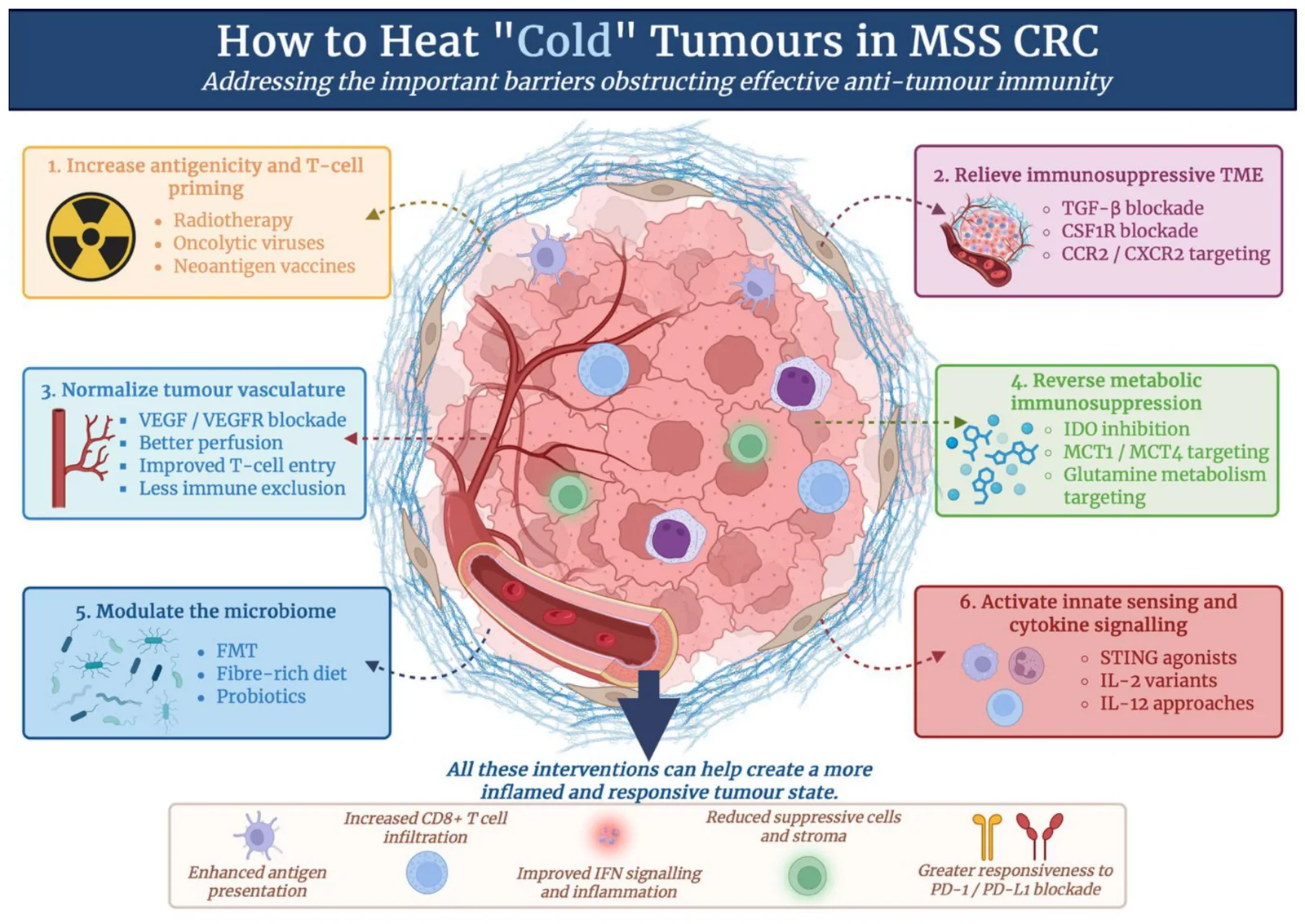
Mechanism-based strategies for converting immunologically “cold” pMMR/MSS colorectal cancer into a more immune-responsive state. These approaches aim to enhance antigen release and presentation, improve T-cell infiltration and function, and reduce stromal, vascular, metabolic and immune-mediated suppression to increase sensitivity to immune checkpoint blockade. Created in BioRender. Roshan, D. (2026) https://BioRender.com/ost0sni.

Developing immunomodulatory strategies have the ability to activate innate sensing and heighten effector immunity, which may help with checkpoint responsiveness. This is further explained in Table 1. Stimulators of interferon response cyclic GMP-AMP (cGAMP) interactor 1 (STING1) agonists are known to trigger type 1 interferon signalling and increase the priming of dendritic cells. They also stimulate the recruitment of CD8+ T-cells, which provides a mechanistic rationale for combination with checkpoint blockade [93]. However, important challenges such as their inconsistent clinical activity and overall accessibility to the tumour site remain as concerns [94,95]. Cytokine therapies have been undergoing significant remodelling in hopes of widening the therapeutic window. Conventional interleukin-2 (IL-2) sometimes leads to signalling that becomes biased to regulatory T-cells or limited by toxicity. New IL-2 variants are being developed to handle these side effects of conventional IL-2 and potentially increase the number of cytotoxic T-cells and natural killer (NK) cells [96]. Similarly, interleukin-12 (IL-12) is a strong pro-inflammatory cytokine that is capable of raising the production of IFN-γ and increasing the strength of the intratumoral immune activation. Even so, the presence of systemic toxicity has made targeted and local delivery platforms more favourable [97]. Preclinical and early translational studies suggest that these approaches may improve antigen presentation and lymphocyte trafficking and thereby enhance sensitivity to PD-1/PD-L1 blockade. However, evidence specific to MSS CRC still remains limited [93,94,95,96,97,98]. Long-term effects of these benefits, though, will depend on rational combinations and prospective clinical studies doing biomarker-guided patient selection in the future [98]. This is further explained in Figure 3.

## 6. Clinical Translation and Comparative Evidence for Combination Strategies in pMMR/MSS CRC

The strategies discussed in Section 5 provide a mechanistic rationale for overcoming distinct barriers blocking immune responsiveness in pMMR/MSS CRC. However, biological evidence of tumour “heating” does not necessarily translate into meaningful clinical benefits. Therefore, this section compares the clinical evidence of major combination strategies, focusing more on trial design, efficacy, patient selection, and the consistency of benefits across studies. Accordingly, findings from phase I/II and single-arm studies are interpreted as preliminary or signal-generating, whereas greater importance is given to randomized comparative trials when assessing clinical efficacy.

### 6.1. Cytotoxic Priming Strategies: Chemotherapy and Radiotherapy

The use of chemotherapy could improve tumour immunogenicity through immunogenic cell death and enhanced antigen presentation, giving a biological rationale for combination with checkpoint blockade [105,106,107]. Chemo-immunotherapy combinations have produced mixed clinical results in metastatic CRC as they include both encouraging signals and negative primary endpoints in randomized phase II studies. The CheckMate 9X8 study, which used nivolumab and modified FOLFOX (mFOLFOX)/bevacizumab, was not able to significantly improve the primary progression-free survival (PFS) endpoint, with a median PFS of 11.9 months in both groups (hazard ratio (HR) 0.81; *p*-value (*p*) = 0.30). However, later progression-free survival, response rates and duration of response were found to be slightly better with the regimen containing nivolumab [108]. On the other hand, AtezoTRIBE showed an overall progression-free survival benefit with atezolizumab plus FOLFOXIRI/bevacizumab, although the effect was smaller in the pMMR/MSS subgroup [109]. Through endoplasmic reticulum stress and immunogenic cell death (ICD)-associated danger signalling, it is possible for irinotecan to improve tumour immunogenicity. This evidence, however, is not well validated in colorectal cancer applications [110].

As previously discussed in Section 5.1, radiotherapy can increase tumour immunogenicity and promote T-cell priming, supporting its integration with ICIs [111,112,113]. Some preclinical and translational works have shown that radiation has helped to increase the intratumoral infiltration of CD8+ T-cells and interferon-related signalling [114]. A phase II study combining radiotherapy with dual checkpoint blockade reported immune remodelling and preliminary activity, although these findings did not establish comparative efficacy [115]. Despite not having observed regression in any tumours outside of the irradiated site, a randomized trial based on metastatic MSS colorectal cancer found feasibility and immunological changes that were measurable [114]. Radioimmunotherapy therefore remains a biologically promising but investigational strategy in MSS CRC. Whether these immunological effects translate into durable clinical benefit will require further studies addressing dose, fractionation, timing and patient selection.

### 6.2. Targeted and Microenvrionment-Sensitizing Combinations

The inhibition of VEGF may enhance immune-cell trafficking and support combination with checkpoint blockade, which has been discussed in Section 5.3 [116]. Epidermal growth factor receptor (EGFR) inhibition with cetuximab is also immunologically valuable because antibody-dependent cellular cytotoxicity is stimulated with cetuximab, along with an improvement in the recruitment of immune cells [117]. The single-arm phase II CAVE trial reported a signal of clinical activity with cetuximab rechallenge plus avelumab in selected previously treated patients with rat sarcoma viral oncogene homologue (RAS) wild-type metastatic CRC. However, the absence of a randomized comparator limits any conclusions regarding efficacy [118]. Another sensitizing method is mitogen-activated protein kinase kinase (MEK) inhibitors as blocking the MAPK pathways could possibly increase the entry of intratumoral T-cells and improve responsiveness to the inhibition of PD-1. However, their clinical benefits depend entirely on the context behind each patient, and they have not yet been well validated in MSS disease [119]. Similarly, the phase III IMBlaxe370 trial did not produce any improvement in overall survival with the atezolizumab plus cobimetinib compared with regorafenib (median OS 8.87 versus 8.51 months; HR 1.00; *p* = 0.99). Thus, it is understood that a biologically promising combination does not necessarily mean a clear clinical benefit in predominantly MSS metastatic CRC [120].

### 6.3. Checkpoint Intensification and Multimodal Strategies

Dual checkpoint blockade aims to broaden immune activation beyond PD-1 inhibition alone. Phase II CheckMate 142 studies reported long-lasting activity with nivolumab plus low-dose ipilimumab in previously treated and first-line settings [7,8]. Such success, however, could not be achieved in MSS CRC, which has a stronger immune exclusion, lower neoantigenicity and baseline T-cell infiltration [121]. Dual checkpoint blockade alone has shown limited efficacy in unselected MSS CRC. This has provided a rationale for investigating sensitizing approaches such as radiotherapy, chemotherapy and other strategies that may reduce immune suppression [122,123]. A randomized study that combined PD-L1 and CTLA-4 blockade with radiation noticed that there was barely any activity. Through this, it can be understood that MSS tumours often cannot be “heated” with only checkpoint strengthening [114].

**Figure 4:**
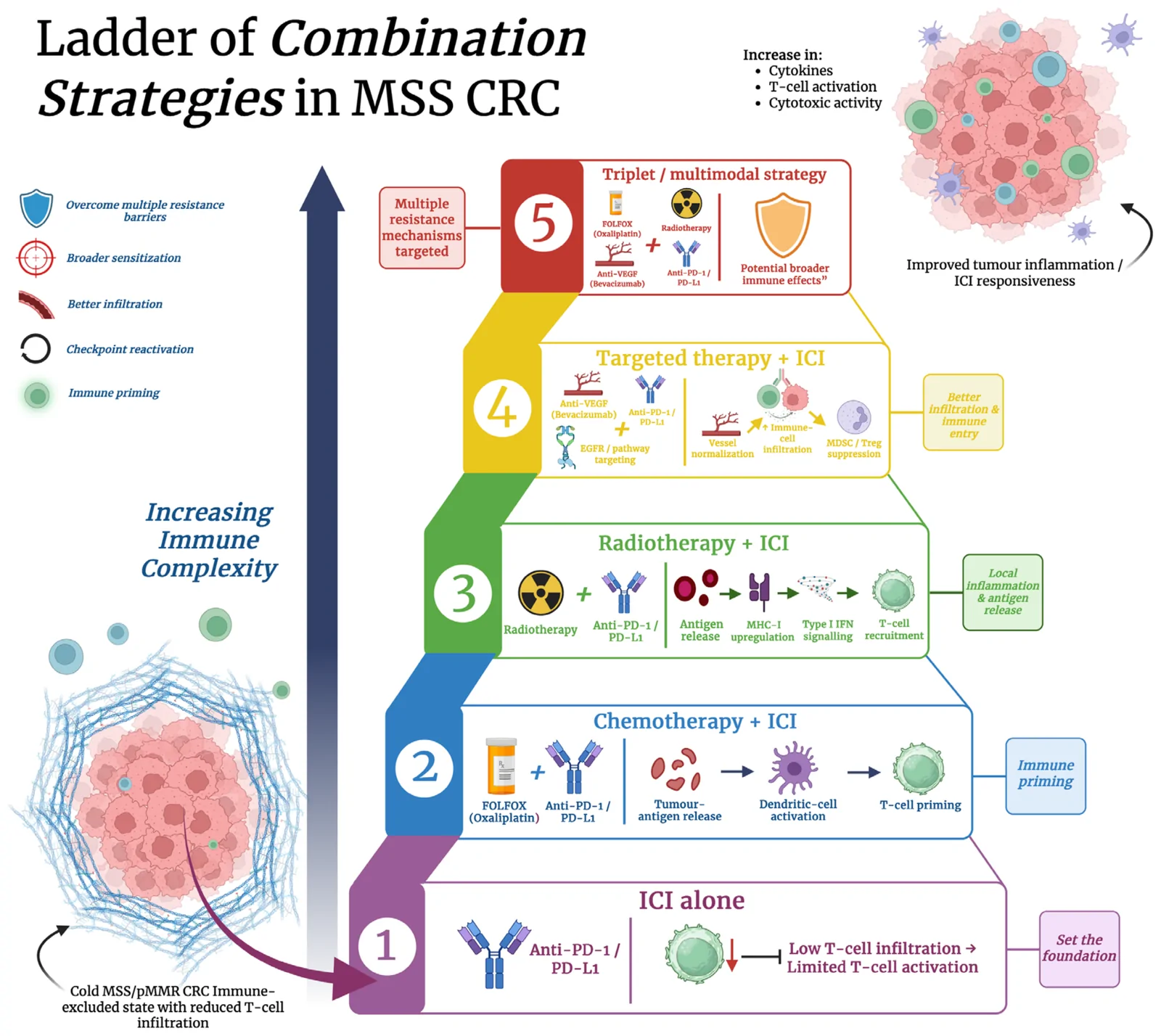
Increasing mechanistic complexity of combination strategies investigated in pMMR/MSS colorectal cancer. Combining immune checkpoint inhibitors with chemotherapy, radiotherapy or targeted therapies may address multiple resistance mechanisms simultaneously. However, greater treatment complexity does not necessarily result in greater clinical benefit, as outcomes remain dependent on tumour biology and patient selection. Created in BioRender. Roshan, D. (2026) https://BioRender.com/jfib05t.

Triplet and multimodal systems are being used to overcome the immune resistance often faced in microsatellite-stable colorectal cancer, which is seen in Figure 4. A good example is the randomized BACCI trial, which used capecitabine and bevacizumab plus atezolizumab. The combination, when used in heavily pretreated metastatic colorectal cancer, had given a slight delay in disease progression when compared with just capecitabine and bevacizumab without any immunotherapy drug. The randomized phase II BACCI trial showed only a modest PFS difference, which was not considered clinically meaningful overall [124]. The MODUL trial is another notable one, which used atezolizumab with fluoropyrimidine/bevacizumab maintenance treatment but could not meet the prespecified PFS hypothesis (HR 0.92; 95% CI 0.72–1.17). It was found that the treatment combination is feasible in practice, but the benefits were seen only in some patients and not in all of them. This highlighted the need for selecting patients on an immune or biomarker basis [125]. A more aggressive multimodal strategy can be seen in the FFCF 1709-SIRTCI trial, which combined liver-directed radioembolization with XELOX, bevacizumab, and atezolizumab [126]. Though there is good biological basis to support such multimodal radioimmunotherapy strategies, it lacks clinical evidence that strongly proves its effectiveness.

Checkpoint inhibitors are increasingly being tested by early-phase studies in combination strategies rather than just as monotherapy. This is a trend seen in the clinical trial field covering MSS colorectal cancer, as shown in Table 2. In a phase I study, botensilimab plus balstilimab showed promising activity in heavily pretreated MSS metastatic CRC, although the uncontrolled design prevents firm conclusions regarding efficacy [127]. Some combinations based on pembrolizumab include the chemotherapy-backed KEYNOTE-651 [128], and the C-C chemokine receptor 5 (CCR5) blockade seen in PICCASSO [22]. In these phase Ib/phase I environments, they produced results that were encouraging, but only observable in selected patients. Also popular are the combinations using ICIs with anti-angiogenic or EGFR-targeted therapies [129]. Results from vicriviroc plus pembrolizumab showed tolerable but limited effects [130], and evidence from tislelizumab with cetuximab and irinotecan had given more reason to explore triplet strategies [131]. The variation in responses is mostly due to the biomarkers and is not completely random. With encorafenib, cetuximab, and nivolumab, positive effects could be observed surrounding the baseline MAPK and immune-activation signatures in MSS B-Raf proto-oncogene, serine/threonine kinase V600E (BRAF^V600E^) disease, whereas the complement pathway activation showed no response [132]. Tumour mutational burden (TMB) that is measured in plasma cannot be assumed as being interchangeable with TMB that was measured in the tumour tissue when identifying patients for immunotherapy [133]. These data, overall, recommend that MSS trials in the future should push towards molecularly different subgroups which are immunologically permissive.

Overall, the intensification of treatments did not consistently improve outcomes in unselected pMMR/MSS CRC. Negative or modest results were seen in CheckMate 9X8, BACCI, MODUL, and IMBlaze370, while AtezoTRIBE had shown an overall benefit with a smaller effect on pMMR/MSS disease. Stronger signals were observed in early-phase or biomarker-selected settings. This shows that rather than adding more therapeutic components, better patient selection and providing treatment according to the dominant resistance mechanism could give a better chance at successfully “heating” the tumour.

## 7. Clinical Translation and Future Research Priorities

### 7.1. Biomarker-Guided Patient Stratification

Selecting patients based on biomarkers in metastatic colorectal cancer remains a difficult task, as apart from dMMR/MSI-H, there is not a single marker that is very reliable. Both PD-L1 expression and TMB in MSS disease have shown limited usability. This could partly be because they do not always represent the functional immune status of the tumour [134,135]. Rather than just looking at PD-L1 or TMB, studying how much the tumour has been infiltrated by active immune cells can provide biomarkers that are more useful for application in immunotherapy [136]. In a single-arm phase II study of pembrolizumab plus azacitidine in chemotherapy-refractory metastatic CRC, higher baseline CD8+ TIL density was associated with a greater likelihood of clinical benefit [137]. In personalized combination strategies, it is crucial to tailor therapies according to the particular tumour environment and not assume that all “cold” tumours are biologically identical. Immune contexture, which involves statistics such as the number, location, and functional status of immune cells, helps to differentiate between various immune patterns. Immune patterns can be inflamed, excluded, or immune-desert, which will determine the most suitable combination of immunotherapy needed for each patient [138,139]. Tools such as Immunoscore and broader immune pathology or transcriptomic signatures are used nowadays to distinguish patients accordingly [140]. Apart from the mismatch repair/microsatellite status, there are not enough robust predictive biomarkers, which creates another challenge. No other marker has yet been consistent enough to be used in routine patient selection. Nevertheless, TMB, immune signatures, and composite biomarker systems seem promising and are currently being studied [141].

### 7.2. Dynamic Monitoring of the “Cold-to-Hot” Transition

The use of dynamic biomarkers has also shown high potential. When MSS metastatic CRC was treated with regorafenib plus PD-1 blockade, an early increase in circulating tumour DNA (ctDNA) levels indicated treatment failure and further progression of the disease. A decrease, though, meant that the disease could temporarily be controlled and stabilized [25]. To help with making the “cold to hot” concept more achievable, imaging can also play a part by providing measurable clinical phenotypes. CD8-directed immune-positron emission tomography (PET) is able to visualize the distribution of T-cells inside the tumour and in the whole body [142]. It provides a dynamic scan of the level of immune infiltration in a non-invasive way, which is often not seen in tissue biopsy alone [143,144]. On a wider scale, more PET, magnetic resonance imaging (MRI), and other related immune-imaging platforms are being developed. These systems will be able to evaluate immune-cell trafficking, treatment response, and the heterogeneity in the tumour which is important to immunotherapy resistance [145]. Radiomics is another modality that provides quantitative imaging features. It can gather information surrounding immune biology, such as the state of the tumour microenvironment and whether there will be any potential responsiveness to the checkpoint blockade [146,147]. Factors such as non-invasive patient stratification and continuous monitoring stand to benefit from these methods.

### 7.3. Optimizing Combination Therapy and Clinical Trial Design

The mixed clinical outcomes that have been described in Section 6 suggest that future development is unlikely to depend on treatment intensification alone, but rather on selecting combinations according to the individual tumour phenotype and the dominant resistance mechanism. Some patients may need VEGF, TGF-β, stromal or myeloid targeted combinations to tackle colorectal cancer, while others may only need an immune checkpoint blockade. In order to advance further into the future, clinical trials must utilize the biomarker results and early response data from the beginning of the treatment and, from there, identify the patients that will benefit from combination treatments [148,149]. Despite there being strong enthusiasm for combination immunotherapy, their transition into everyday MSS CRC treatment has been difficult. One of the biggest issues is the increase in toxicity prior to providing lasting benefits. Trials have suggested that adverse effects seemed to be common in combinations that applied intensive immunotherapies [20]. The AtezoTRIBE trial is one such example that showed this trade-off between positive and adverse effects very well [109]. Clinical progress is further hindered by the fact that MSS CRC is biologically heterogeneous. Differences can occur in immune exclusion, stromal composition, and tumour composition states, which all could affect the sensitivity of treatment [134]. An important factor to be taken into consideration when developing such increasingly complex combination strategies is practical implementation and cost. Accessibility can become an issue due to the high cost of checkpoint inhibitors, particularly when they are combined with additional targeted agents and require prolonged treatment or management of treatment-related toxicity. Financial barriers to the wider implementation of ICIs remain recognized in oncology practice [150]. Biomarker-guided treatment could introduce requirements for specialized molecular testing, new infrastructure and assay standardization. This is a problem as the cost-effectiveness of emerging multi-parameter approaches is yet to be established [151]. In pMMR/MSS CRC, where the clinical benefit of many intensive combinations remains uncertain, these considerations are especially important.

### 7.4. Emerging Therapeutic and Analytical Platforms

Artificial intelligence (AI)-based pathology and machine-learning (ML) models are emerging analytical tools. They can provide well-tuned treatment by combining immune contexture, gene-expression data, and conventional biomarkers, which may even outperform single-marker strategies in deciding appropriate immunotherapy [152]. More specifically, AI-driven digital pathology can identify dMMR/MSI-H-associated histological patterns directly from routine hematoxylin and eosin slides. Multimodal AI approaches can be used to integrate pathology, imaging and molecular data to support immune phenotyping and patient stratification. These tools can help with prioritizing patients for confirmatory tests or identifying features that are relevant to immunotherapy response. However, most AI models still require broader prospective validation. Concerns remain regarding generalizability across centres, interpretability, dataset variability and regulatory integration in routine clinical decision-making for pMMR/MSS CRC patients today [153]. Nanomedicine has also shown strong, applicable potential. Poly(lactic-co-glycolic acid) (PLGA) systems and other such targeted carriers have given improvements in tumour exposure and a reduction in off-target toxicity [154]. They deliver necessary antigens, adjuvants, or checkpoint-directed payloads straight into the colorectal tumour microenvironment [155]. Nanomedicine could be used to complement immunotherapy by improving the intratumoral delivery of immunomodulatory agents, which could, in turn, enhance antigen presentation and allow for the co-delivery of complementary therapeutic payloads. Such approaches are particularly relevant to “cold” CRC because they can remodel the suppressive components of the tumour microenvironment while also limiting systemic exposure. However, most nano-immunotherapy evidence in CRC is preclinical and its translation is hindered by issues such as pharmacokinetic variability, biosafety concerns, manufacturing and scale-up requirements, and regulatory complexity [154,156]. Nanomedicine should therefore be regarded as investigational rather than as an established strategy to overcome immunotherapy resistance in pMMR/MSS. Adding to the options of therapies for CRC are next-generation immunotherapies such as bispecific antibodies and adoptive cell therapies like chimeric antigen receptor T-cell (CAR-T) therapy. Regarding these therapies, though, some challenges still remain in the solid tumours. They include trafficking barriers, antigen heterogeneity, and the immunosuppressive microenvironment [157]. More advanced treatments such as CAR-T therapy have complex manufacturing, quality-control and logistical requirements that currently hinder their scalability and accessibility [158]. As a whole, these future directions push for more personalized and multimodal therapies.

### 7.5. Translational Priorities

Future progress in pMMR/MSS CRC is more likely to depend on mechanism-guided therapy rather than uniformly intensified combination therapy. Patients should be grouped according to the dominant immune-resistance phenotype, with their treatment selected to address deficient priming, immune exclusion, stromal or vascular barriers, or other relevant mechanisms. To determine whether or not the given treatment has produced the “cold to hot” effects, future trials must integrate baseline biomarkers with longitudinal measures such as ctDNA and immune imaging. Therefore, clinical benefits must be interpreted with biological evidence of target engagement and immune remodelling. Such an approach could help to differentiate genuinely effective combinations from those that show compelling mechanistic rationale but clinically have limited benefits.

## 8. Discussion

Overall, the evidence reviewed suggests that immunotherapy resistance in pMMR/MSS colorectal cancer results from several interacting mechanisms, including low tumour antigenicity, impaired T-cell infiltration, stromal and vascular exclusion, immunosuppressive cells and metabolic dysfunction. Although many combination strategies have shown promising biological or early clinical activity, randomized trials have generally demonstrated limited or inconsistent benefit in unselected pMMR/MSS populations [108,109,120,124,125]. These findings suggest that successful treatment may depend more on selecting patients according to their dominant resistance mechanism than on simply intensifying therapy.

The available evidence has several limitations. Many studies were preclinical, translational, early-phase or single-arm investigations, and considerable heterogeneity existed in patient populations, interventions, biomarkers and reported outcomes. This limits direct comparison between studies and prevents firm conclusions regarding several emerging strategies. The review itself was also limited to PubMed and PMC, English-language full-text publications, and a narrative synthesis because the included studies were too heterogeneous for meta-analysis. Formal risk-of-bias, reporting-bias and GRADE certainty assessments were not performed.

For clinical practice, current evidence does not support routine use of experimental ICI combinations in unselected pMMR/MSS CRC. Future trials should prioritize biomarker-guided patient selection, prospective validation of promising combinations, and longitudinal measures such as ctDNA and immune imaging to confirm whether meaningful immune remodelling has occurred.

## 9. Conclusions

The concept of “hot” dMMR/MSI-H and “cold” pMMR/MSS tumours provides a useful framework for understanding the resistance to immunotherapies in colorectal cancer. Treatment options like checkpoint inhibitors have changed the way MSI-H disease is being treated nowadays. However, even with such developments, MSS colorectal cancer can still show poor responsiveness. This is mainly because of certain barriers such as the lack of strong neoantigen presentation, adequate T-cell infiltration, or a supporting immune microenvironment. Tumour intrinsic pathways such as Wnt/β-catenin and MAPK signalling, along with abnormal vasculature, suppressive myeloid cells, and metabolic stress, also play a part in the resistance to immunotherapy. As such, checkpoint blockade alone is generally insufficient in unselected pMMR/MSS CRC treatment. The main idea in treatment should be to convert “cold” tumours into “hot” ones that are inflamed and T-cell-accessible so that an effective response can be seen during PD-1, PD-L1, or CTLA-4 inhibition. Strategies such as chemotherapy, radiotherapy, vascular and myeloid targeting, metabolic reprogramming and microbiome modulation can promote a more inflamed and T-cell-accessible tumour state. However, mixed results from clinical trials show that biological evidence of tumour “heating” does not necessarily translate into meaningful clinical benefit, which is particularly true with pMMR/MSS populations. Future progress should thus place importance on biomarker- and mechanism-guided patient selection rather than uniform treatment intensification. ctDNA and immune imaging are longitudinal approaches that could help to determine whether immune remodelling has actually occurred during treatment. Emerging artificial intelligence and nanomedicine approaches may improve patient stratification and therapeutic precision, although their clinical utility in pMMR/MSS CRC remains under investigation. Ultimately, converting “cold” MSS CRC into clinically responsive disease will depend on matching individual resistance mechanisms with rational and appropriately selected combination strategies.

## Figures and Tables

**Table 1:** Mechanism-based strategies that can be used to overcome resistance and convert immunologically “cold” tumours into “hot” tumours.

Strategy Class	Example Interventions	Main “Heating” Mechanism	Expected Immune Effect	Evidence Stage in pMMR/MSS CRC	Representative Evidence/Key Limitation
Restore antigen release and T-cell priming [99]	Radiotherapy, oncolytic viruses, and neoantigen vaccines	Increases the death of immunogenic cells and tumour-antigen presentation	Better activation of dendritic cells and CD8+ T-cell priming	Preclinical/translational to phase II clinical evidence	Clinical activity is mixed, with limited benefit in randomized MSS CRC studies [99].
Reverse immunosuppressive cellular and stromal signalling [100]	TGF-β blockade, CSF1R, CCR2, and CXCR2-guided myeloid targeting	Reduces immune exclusion and suppressive stromal and myeloid signalling	An improvement in T-cell infiltration and less myeloid-mediated suppression	Predominantly preclinical and translational evidence	Strong mechanistic evidence, but limited clinical validation in MSS CRC [70,71,72,73].
Normalize tumour vasculature and restore immune trafficking [101]	VEGF or VEGFR inhibition, particularly with ICIs	Improves vascular function and the trafficking of immune cells	Better lymphocyte entry and better checkpoint response	Preclinical/translational to randomized phase II clinical evidence	Clinical results remain inconsistent across anti-VEGF/ICI combinations [101].
Reverse metabolic and hypoxic immunosuppression [79,102]	IDO inhibitors, MCT1 or 4 inhibition, and glutamine-targeting approaches	Lowers the metabolic barriers that blunt effector immunity	Improves the fitness of T-cells and reduces the suppressive metabolite pressure	Predominantly preclinical evidence with limited clinical validation	Promising preclinical evidence, but limited MSS CRC clinical validation [81,82,83,84,85].
Modulate microbiome-associated resistance [103]	FMT, diet, and probiotics	Remodels systemic and gut-derived immune signalling	May improve the overall responsiveness to ICIs in certain environments	Early clinical evidence, limited MSS CRC-specific validation	Promising single-arm MSS CRC data, but randomized validation is lacking [88,89]
Activate innate immune sensing and cytokine signalling [104]	STING agonists, IL-2 variants and IL-12 approaches	Boosts the activation of innate immune and pro-inflammatory cytokine tone	Converts previously poorly inflamed tumours into T-cell-inflamed states	Preclinical to early clinical evidence, predominantly outside MSS CRC	Early clinical development continues, but MSS CRC-specific evidence remains limited [93,94,95,96,97,98].

Evidence stage is based on the development level of available evidence and does not represent a formal GRADE assessment. Representative evidence was drawn from studies cited in this review and limited pMMR/MSS-specific evidence is indicated where applicable. Abbreviations: C-C chemokine receptor 2 (CCR2), colorectal cancer (CRC), colony-stimulating factor 1 receptor (CSF1R), C-X-C chemokine receptor 2 (CXCR2), fecal microbiota transplantation (FMT), immune checkpoint inhibitor (ICI), indoleamine 2,3-dioxygenase (IDO), interleukin (IL), monocarboxylate transporter (MCT), microsatellite stable (MSS), proficient mismatch repair (pMMR), stimulator of interferon genes (STING), transforming growth factor-beta (TGF-β), vascular endothelial growth factor (VEGF), and vascular endothelial growth factor receptor (VEGFR).

**Table 2:** Notable combination strategies used in pMMR/MSS colorectal cancer.

Combination Strategy	Representative Study and Design	Population	Key Efficacy Findings	Overall Interpretation
Chemotherapy + anti-PD-1 + anti-VEGF [108]	CheckMate 9X8, randomized phase II	First-line unresectable mCRC, n = 195 randomized	PFS 11.9 vs. 11.9 months (HR 0.81; *p* = 0.30), ORR 60% vs. 46%.	Primary endpoint negative, despite having favourable response signals.
Chemotherapy + anti-PD-L1 + anti-VEGF [109]	AtezoTRIBE, randomized phase II	Previously untreated mCRC, n = 218; predominantly pMMR/MSS	PFS 13.1 vs. 11.5 months (HR 0.69; *p* = 0.012), smaller benefit in pMMR/MSS disease.	Positive overall, but benefit in pMMR/MSS was more limited.
Radiotherapy + anti-PD-L1 + anti-CTLA-4 [114]	Monjazeb et al.; randomized phase II	Heavily pretreated metastatic MSS CRC, 18 patients evaluable for response	No objective responses outside the radiation field, median OS 3.8 months.	Limited clinical activity despite immune remodelling.
Chemotherapy + anti-VEGF + anti-PD-L1 [124]	BACCI, randomized, double-blind phase II	Refractory mCRC, n = 128 efficacy-evaluable, including 110 MSS patients	PFS 4.4 vs. 3.6 months (HR 0.75), MSS subgroup HR 0.66.	Modest benefit, not clinically meaningful overall.
Maintenance anti-VEGF + anti-PD-L1 [125]	MODUL cohort 2, randomized signal-seeking trial	First-line BRAF-wild-type mCRC after FOLFOX/bevacizumab induction, n = 445	No PFS improvement (HR 0.92; *p* = 0.48).	Negative randomized trial.
Enhanced CTLA-4 + anti-PD-1 [127]	Botensilimab + balstilimab, phase I	Heavily pretreated MSS mCRC, n = 148; 101 response-evaluable	ORR 17%; DCR 61%, median PFS 3.5 months.	Promising phase I activity, but uncontrolled.
BRAF + EGFR + anti-PD-1 [132]	Encorafenib + cetuximab + nivolumab, phase I/II	Biomarker-selected MSS BRAF V600E mCRC, n = 26	ORR 50%, median PFS 7.4 months.	Promising biomarker-selected activity, but based on a small non-randomized study.
MEK inhibition + anti-PD-L1 [120]	IMblaze370, randomized phase III	Previously treated predominantly MSS metastatic CRC, n = 363	OS 8.87 vs. 8.51 months (HR 1.00; *p* = 0.99).	Negative phase III trial.

Representative prospective trials of ICI-based combinations in pMMR/MSS metastatic CRC are shown, including positive, mixed, and negative outcomes. Efficacy data were taken from the cited primary studies, and their interpretations are descriptive and do not represent formal GRADE assessment. Abbreviations: Confidence interval (CI), disease control rate (DCR), hazard ratio (HR), immune checkpoint inhibitor (ICI), metastatic colorectal cancer (mCRC), microsatellite stable (MSS), objective response rate (ORR), overall survival (OS), progression-free survival (PFS), and proficient mismatch repair (pMMR). Early-phase and single-arm studies are interpreted as signal-generating rather than confirmatory evidence of efficacy.

## Data Availability

No new data were created or analyzed in this study. Data sharing is not applicable to this article.

## Supplementary Materials

The following supporting information can be downloaded at: https://www.mdpi.com/article/10.3390/molecules31173124/s1.
