# Supplementary material for: Heating the Cold: Overcoming Immunotherapy Resistance in Microsatellite-Stable Colorectal Cancer: A Systematic Review
Source: Molecules. 2026 Sep 6;31(17):3124. doi: 10.3390/molecules31173124 (PMC13567396; doi:10.3390/molecules31173124)
Supplement: Supplementary file 1 [file molecules-31-03124-s001.zip › molecules-4492843-supplementary.pdf]

PRISMA 2020 Checklist

Table S1

Section and Topic	Item #	Checklist item	Location where item is reported
**TITLE**			
Title	1	Identify the report as a systematic review.	Page 1
**ABSTRACT**			
Abstract	2	See the PRISMA 2020 for Abstracts checklist.	Page 1
**INTRODUCTION**			
Rationale	3	Describe the rationale for the review in the context of existing knowledge.	Page 4
Objectives	4	Provide an explicit statement of the objective(s) or question(s) the review addresses.	Page 4
**METHODS**			
Eligibility criteria	5	Specify the inclusion and exclusion criteria for the review and how studies were grouped for the syntheses.	Page 5
Information sources	6	Specify all databases, registers, websites, organisations, reference lists and other sources searched or consulted to identify studies. Specify the date when each source was last searched or consulted.	Page 5
Search strategy	7	Present the full search strategies for all databases, registers and websites, including any filters and limits used.	Supplementary Material S2
Selection process	8	Specify the methods used to decide whether a study met the inclusion criteria of the review, including how many reviewers screened each record and each report retrieved, whether they worked independently, and if applicable, details of automation tools used in the process.	Page 5
Data collection process	9	Specify the methods used to collect data from reports, including how many reviewers collected data from each report, whether they worked independently, any processes for obtaining or confirming data from study investigators, and if applicable, details of automation tools used in the process.	Page 5
Data items	10a	List and define all outcomes for which data were sought. Specify whether all results that were compatible with each outcome domain in each study were sought (e.g. for all measures, time points, analyses), and if not, the methods used to decide which results to collect.	Page 5
	10b	List and define all other variables for which data were sought (e.g. participant and intervention characteristics, funding sources). Describe any assumptions made about any missing or unclear information.	Page 5
Study risk of bias assessment	11	Specify the methods used to assess risk of bias in the included studies, including details of the tool(s) used, how many reviewers assessed each study and whether they worked independently, and if applicable, details of automation tools used in the process.	Page 5
Effect measures	12	Specify for each outcome the effect measure(s) (e.g. risk ratio, mean difference) used in the synthesis or presentation of results.	Page 5
Synthesis methods	13a	Describe the processes used to decide which studies were eligible for each synthesis (e.g. tabulating the study intervention characteristics and comparing against the planned groups for each synthesis (item #5)).	Page 5
	13b	Describe any methods required to prepare the data for presentation or synthesis, such as handling of missing summary statistics, or data conversions.	Page 5
	13c	Describe any methods used to tabulate or visually display results of individual studies and syntheses.	Page 5
	13d	Describe any methods used to synthesize results and provide a rationale for the choice(s). If meta-analysis was performed, describe the model(s), method(s) to identify the presence and extent of statistical heterogeneity, and software package(s) used.	Page 5
	13e	Describe any methods used to explore possible causes of heterogeneity among study results (e.g. subgroup analysis, meta-regression).	Page 5
	13f	Describe any sensitivity analyses conducted to assess robustness of the synthesized results.	Page 5

PRISMA 2020 Checklist

Section and Topic	Item #	Checklist item	Location where item is reported
Reporting bias assessment	14	Describe any methods used to assess risk of bias due to missing results in a synthesis (arising from reporting biases).	Page 5
Certainty assessment	15	Describe any methods used to assess certainty (or confidence) in the body of evidence for an outcome.	Page 5
**RESULTS**			
Study selection	16a	Describe the results of the search and selection process, from the number of records identified in the search to the number of studies included in the review, ideally using a flow diagram.	Page 6
	16b	Cite studies that might appear to meet the inclusion criteria, but which were excluded, and explain why they were excluded.	Supplementary Material S3
Study characteristics	17	Cite each included study and present its characteristics.	Supplementary Material S4
Risk of bias in studies	18	Present assessments of risk of bias for each included study.	Page 6
Results of individual studies	19	For all outcomes, present, for each study: (a) summary statistics for each group (where appropriate) and (b) an effect estimate and its precision (e.g. confidence/credible interval), ideally using structured tables or plots.	Supplementary Material S4
Results of syntheses	20a	For each synthesis, briefly summarise the characteristics and risk of bias among contributing studies.	Page 6
	20b	Present results of all statistical syntheses conducted. If meta-analysis was done, present for each the summary estimate and its precision (e.g. confidence/credible interval) and measures of statistical heterogeneity. If comparing groups, describe the direction of the effect.	Page 6
	20c	Present results of all investigations of possible causes of heterogeneity among study results.	Page 6
	20d	Present results of all sensitivity analyses conducted to assess the robustness of the synthesized results.	Page 6
Reporting biases	21	Present assessments of risk of bias due to missing results (arising from reporting biases) for each synthesis assessed.	Page 6
Certainty of evidence	22	Present assessments of certainty (or confidence) in the body of evidence for each outcome assessed.	Page 6
**DISCUSSION**			
Discussion	23a	Provide a general interpretation of the results in the context of other evidence.	Page 22
	23b	Discuss any limitations of the evidence included in the review.	Page 22
	23c	Discuss any limitations of the review processes used.	Page 22
	23d	Discuss implications of the results for practice, policy, and future research.	Page 22
**OTHER INFORMATION**			
Registration and protocol	24a	Provide registration information for the review, including register name and registration number, or state that the review was not registered.	Page 5
	24b	Indicate where the review protocol can be accessed, or state that a protocol was not prepared.	Page 5
	24c	Describe and explain any amendments to information provided at registration or in the protocol.	Page 5
Support	25	Describe sources of financial or non-financial support for the review, and the role of the funders or sponsors in the review.	Page 23
Competing interests	26	Declare any competing interests of review authors.	Page 23
Availability of	27	Report which of the following are publicly available and where they can be found: template data collection forms; data extracted from included	Page 6

Section and Topic	Item #	Checklist item	Location where item is reported
data, code and other materials		studies; data used for all analyses; analytic code; any other materials used in the review.	

From: Page MJ, McKenzie JE, Bossuyt PM, Boutron I, Hoffmann TC, Mulrow CD, et al. The PRISMA 2020 statement: an updated guideline for reporting systematic reviews. BMJ 2021;372:n71. doi: 10.1136/bmj.n71. This work is licensed under CC BY 4.0. To view a copy of this license, visit https://creativecommons.org/licenses/by/4.0/

Table S2

Database	Search strategy
**PubMed – Search 1**	**Query:** colorectal cancer immunotherapy**Search Details:** ("colorectal neoplasms"[MeSH Terms] OR ("colorectal"[All Fields] AND "neoplasms"[All Fields]) OR "colorectal neoplasms"[All Fields] OR ("colorectal"[All Fields] AND "cancer"[All Fields]) OR "colorectal cancer"[All Fields]) AND ("immunotherapy"[MeSH Terms] OR "immunotherapy"[All Fields] OR "immunotherapies"[All Fields] OR "immunotherapy s"[All Fields])**Filters:** None
**PubMed – Search 2**	**Query:** colorectal cancer immunotherapy resistance**Search Details:** ("colorectal neoplasms"[MeSH Terms] OR ("colorectal"[All Fields] AND "neoplasms"[All Fields]) OR "colorectal neoplasms"[All Fields] OR ("colorectal"[All Fields] AND "cancer"[All Fields]) OR "colorectal cancer"[All Fields]) AND ("immunotherapy"[MeSH Terms] OR "immunotherapy"[All Fields] OR "immunotherapies"[All Fields] OR "immunotherapy s"[All Fields]) AND ("resist"[All Fields] OR "resistance"[All Fields] OR "resistances"[All Fields] OR "resistant"[All Fields] OR "resistants"[All Fields] OR "resisted"[All Fields] OR "resistence"[All Fields] OR "resistences"[All Fields] OR "resistent"[All Fields] OR "resistibility"[All Fields] OR "resisting"[All Fields] OR "resistive"[All Fields] OR "resistively"[All Fields] OR "resistivities"[All Fields] OR "resistivity"[All Fields] OR "resists"[All Fields])**Filters:** None
**PubMed – Search 3**	**Query:** microsatellite stable colorectal cancer immunotherapy**Search Details:** ("microsatellite repeats"[MeSH Terms] OR ("microsatellite"[All Fields] AND "repeats"[All Fields]) OR "microsatellite repeats"[All Fields] OR "microsatellite"[All Fields] OR "microsatellites"[All Fields]) AND ("stable"[All Fields] OR "stabled"[All Fields] OR "stables"[All Fields] OR "stabling"[All Fields]) AND ("colorectal neoplasms"[MeSH Terms] OR ("colorectal"[All Fields] AND "neoplasms"[All Fields]) OR "colorectal neoplasms"[All Fields] OR ("colorectal"[All Fields] AND "cancer"[All Fields]) OR "colorectal cancer"[All Fields]) AND ("immunotherapy"[MeSH Terms] OR "immunotherapy"[All Fields] OR "immunotherapies"[All Fields] OR "immunotherapy s"[All Fields])**Filters:**None
**PubMed – Search 4**	**Query:** MSS colorectal cancer immune checkpoint inhibitors**Search Details:** "MSS"[All Fields] AND ("colorectal neoplasms"[MeSH Terms] OR ("colorectal"[All Fields] AND "neoplasms"[All Fields]) OR "colorectal neoplasms"[All Fields] OR ("colorectal"[All Fields] AND "cancer"[All Fields]) OR "colorectal cancer"[All Fields]) AND ("immune checkpoint inhibitors"[Pharmacological Action] OR "immune checkpoint inhibitors"[Supplementary Concept] OR "immune checkpoint inhibitors"[All Fields] OR "immune checkpoint inhibitors"[MeSH Terms] OR ("immune"[All Fields] AND "checkpoint"[All Fields] AND "inhibitors"[All Fields]))**Filters:** None

**PubMed – Search 5**	**Query:** pMMR colorectal cancer immunotherapy**Search Details:** "pMMR"[All Fields] AND ("colorectal neoplasms"[MeSH Terms] OR ("colorectal"[All Fields] AND "neoplasms"[All Fields]) OR "colorectal neoplasms"[All Fields] OR ("colorectal"[All Fields] AND "cancer"[All Fields]) OR "colorectal cancer"[All Fields]) AND ("immunotherapy"[MeSH Terms] OR "immunotherapy"[All Fields] OR "immunotherapies"[All Fields] OR "immunotherapy s"[All Fields])**Filters:** None
**PubMed – Search 6**	**Query:** colorectal cancer immune checkpoint blockade resistance**Search Details:** ("colorectal neoplasms"[MeSH Terms] OR ("colorectal"[All Fields] AND "neoplasms"[All Fields]) OR "colorectal neoplasms"[All Fields] OR ("colorectal"[All Fields] AND "cancer"[All Fields]) OR "colorectal cancer"[All Fields]) AND ("immune checkpoint inhibitors"[Pharmacological Action] OR "immune checkpoint inhibitors"[Supplementary Concept] OR "immune checkpoint inhibitors"[All Fields] OR "immune checkpoint blockade"[All Fields] OR "immune checkpoint inhibitors"[MeSH Terms] OR ("immune"[All Fields] AND "checkpoint"[All Fields] AND "inhibitors"[All Fields]) OR ("immune"[All Fields] AND "checkpoint"[All Fields] AND "blockade"[All Fields])) AND ("resist"[All Fields] OR "resistance"[All Fields] OR "resistances"[All Fields] OR "resistant"[All Fields] OR "resistants"[All Fields] OR "resisted"[All Fields] OR "resistence"[All Fields] OR "resistences"[All Fields] OR "resistent"[All Fields] OR "resistibility"[All Fields] OR "resisting"[All Fields] OR "resistive"[All Fields] OR "resistively"[All Fields] OR "resistivities"[All Fields] OR "resistivity"[All Fields] OR "resists"[All Fields])**Filters:** None
**PubMed – Search 7**	**Query:** cold colorectal cancer immunotherapy**Search Details:** ("common cold"[MeSH Terms] OR ("common"[All Fields] AND "cold"[All Fields]) OR "common cold"[All Fields] OR "cold"[All Fields] OR "cold temperature"[MeSH Terms] OR ("cold"[All Fields] AND "temperature"[All Fields]) OR "cold temperature"[All Fields]) AND ("colorectal neoplasms"[MeSH Terms] OR ("colorectal"[All Fields] AND "neoplasms"[All Fields]) OR "colorectal neoplasms"[All Fields] OR ("colorectal"[All Fields] AND "cancer"[All Fields]) OR "colorectal cancer"[All Fields]) AND ("immunotherapy"[MeSH Terms] OR "immunotherapy"[All Fields] OR "immunotherapies"[All Fields] OR "immunotherapy s"[All Fields])**Filters:** None
**PubMed – Search 8**	**Query:** colorectal cancer immune exclusion**Search Details:** ("colorectal neoplasms"[MeSH Terms] OR ("colorectal"[All Fields] AND "neoplasms"[All Fields]) OR "colorectal neoplasms"[All Fields] OR ("colorectal"[All Fields] AND "cancer"[All Fields]) OR "colorectal cancer"[All Fields]) AND ("immune"[All Fields] OR "immuned"[All Fields] OR "immunes"[All Fields] OR "immunisation"[All Fields] OR "vaccination"[MeSH Terms] OR "vaccination"[All Fields] OR "immunization"[All Fields] OR "immunization"[MeSH Terms] OR "immunisations"[All Fields] OR "immunizations"[All Fields] OR "immunise"[All Fields] OR "immunised"[All Fields] OR "immuniser"[All Fields] OR "immunisers"[All Fields] OR "immunising"[All Fields] OR "immunities"[All Fields] OR "immunity"[MeSH Terms] OR "immunity"[All Fields] OR "immunization s"[All Fields] OR "immunize"[All Fields] OR "immunized"[All Fields] OR "immunizer"[All Fields] OR "immunizers"[All Fields] OR "immunizes"[All Fields] OR "immunizing"[All Fields]) AND ("exclusion"[All Fields] OR "exclusions"[All Fields])**Filters:** None
**PubMed – Search 9**	**Query:** colorectal cancer tumor microenvironment immunotherapy**Search Details:** ("colorectal neoplasms"[MeSH Terms] OR ("colorectal"[All Fields] AND "neoplasms"[All Fields]) OR "colorectal neoplasms"[All Fields] OR ("colorectal"[All Fields] AND "cancer"[All Fields]) OR "colorectal cancer"[All Fields]) AND ("tumour microenvironment"[All Fields] OR "tumor microenvironment"[MeSH Terms] OR ("tumor"[All Fields] AND "microenvironment"[All Fields]) OR "tumor microenvironment"[All Fields]) AND ("immunotherapy"[MeSH Terms] OR "immunotherapy"[All Fields] OR "immunotherapies"[All Fields] OR "immunotherapy s"[All Fields])**Filters:** None
**PubMed – Search 10**	**Query:** colorectal cancer PD-1 PD-L1 resistance**Search Details:** ("colorectal neoplasms"[MeSH Terms] OR ("colorectal"[All Fields] AND "neoplasms"[All Fields]) OR "colorectal neoplasms"[All Fields] OR ("colorectal"[All Fields] AND "cancer"[All Fields]) OR "colorectal cancer"[All Fields]) AND ("pdcd1 protein

	human"[Supplementary Concept] OR "pdcd1 protein human"[All Fields] OR "pd 1"[All Fields]) AND ("cd274 protein human"[Supplementary Concept] OR "cd274 protein human"[All Fields] OR "pd l1"[All Fields]) AND ("resist"[All Fields] OR "resistance"[All Fields] OR "resistances"[All Fields] OR "resistant"[All Fields] OR "resistants"[All Fields] OR "resisted"[All Fields] OR "resistence"[All Fields] OR "resistences"[All Fields] OR "resistent"[All Fields] OR "resistibility"[All Fields] OR "resisting"[All Fields] OR "resistive"[All Fields] OR "resistively"[All Fields] OR "resistivities"[All Fields] OR "resistivity"[All Fields] OR "resists"[All Fields]) **Filters:** None

Database	Search strategy
PMC	"pMMR"[All Fields] AND ("colorectal neoplasms"[MeSH Terms] OR ("colorectal"[All Fields] AND "neoplasms"[All Fields]) OR "colorectal neoplasms"[All Fields] OR ("colorectal"[All Fields] AND "cancer"[All Fields]) OR "colorectal cancer"[All Fields]) AND ("immune checkpoint inhibitors"[All Fields] OR "immune checkpoint inhibitors"[Supplementary Concept] OR "immune checkpoint inhibitors"[All Fields] OR "immune checkpoint inhibitors"[MeSH Terms] OR ("immune"[All Fields] AND "checkpoint"[All Fields] AND "inhibitors"[All Fields]))

Table S3

Study citation	Reason for exclusion
André T, Shiu KK, Kim TW, et al. *Pembrolizumab versus chemotherapy in microsatellite instability-high or mismatch repair-deficient metastatic colorectal cancer: 5-year follow-up from the randomized phase III KEYNOTE-177 study.* Ann Oncol. 2025;36(3):277–284. PMID: 39631622.	**Not related to pMMR/MSS CRC:** study population was MSI-H/dMMR metastatic CRC.
Andre T, Elez E, Van Cutsem E, et al. *Nivolumab plus Ipilimumab in Microsatellite-Instability-High Metastatic Colorectal Cancer.* N Engl J Med. 2024;391(21):2014–2026. PMID: 39602630.	**Not related to pMMR/MSS CRC:** restricted to MSI-H/dMMR metastatic CRC.
Cohen R, Bennouna J, Meurisse A, et al. *RECIST and iRECIST criteria for the evaluation of nivolumab plus ipilimumab in patients with microsatellite instability-high/mismatch repair-deficient metastatic colorectal cancer: the GERCOR NIPICOL phase II study.* J Immunother Cancer. 2020;8(2):e001499. PMID: 33148693.	**Not related to pMMR/MSS CRC:** evaluated MSI-H/dMMR metastatic CRC.
Cercek A, Foote MB, Rousseau B, et al. *Nonoperative Management of Mismatch Repair-Deficient Tumors.* N Engl J Med. 2025;392(23):2297–2308. PMID: 40293177.	**Not related to pMMR/MSS CRC:** investigated mismatch repair-deficient tumours rather than pMMR/MSS CRC.
Overman MJ, Gelsomino F, Aglietta M, et al. *Nivolumab plus relatlimab in patients with previously treated microsatellite instability-high/mismatch repair-deficient metastatic colorectal cancer: the phase II CheckMate 142 study.* J Immunother Cancer. 2024;12(5):e008689. PMID: 38821718.	**Not related to pMMR/MSS CRC:** population was MSI-H/dMMR metastatic CRC.
Cercek A, Lumish M, Sinopoli J, et al. *PD-1 Blockade in Mismatch Repair-Deficient, Locally Advanced Rectal Cancer.* N Engl J Med. 2022;386(25):2363–2376. PMID: 35660797.	**Not related to pMMR/MSS CRC:** included only mismatch repair-deficient rectal cancer.
Le DT, Diaz LA Jr, Kim TW, et al. *Pembrolizumab for previously treated, microsatellite instability-high/mismatch repair-deficient advanced colorectal cancer: final analysis of*	**Not related to pMMR/MSS CRC:** study concerned MSI-H/dMMR advanced CRC.

KEYNOTE-164. Eur J Cancer. 2023;186:185–195. PMID: 37141828.	
Gögenur I, Justesen TF, Tarpgaard LS, et al. *Neoadjuvant Pembrolizumab in Stages I-III Deficient Mismatch Repair Colon Cancer: A Clinical Trial.* Ann Surg. 2026;283(6):1075–1081. PMID: 39692004.	**Not related to pMMR/MSS CRC:**restricted to dMMR colon cancer.
André T, Elez E, Lenz HJ, et al. *Nivolumab plus ipilimumab versus nivolumab in microsatellite instability-high metastatic colorectal cancer (CheckMate 8HW).*Lancet. 2025;405(10476):383–395. PMID: 39874977.	**Not related to pMMR/MSS CRC:**evaluated MSI-H metastatic CRC rather than MSS/pMMR disease.
Diaz LA Jr, Shiu KK, Kim TW, et al. *Pembrolizumab versus chemotherapy for microsatellite instability-high or mismatch repair-deficient metastatic colorectal cancer (KEYNOTE-177): final analysis.* Lancet Oncol. 2022;23(5):659–670. PMID: 35427471.	**Not related to pMMR/MSS CRC:**population was MSI-H/dMMR metastatic CRC.
Le DT, Kim TW, Van Cutsem E, et al. *Phase II Open-Label Study of Pembrolizumab in Treatment-Refractory, Microsatellite Instability-High/Mismatch Repair-Deficient Metastatic Colorectal Cancer: KEYNOTE-164.*J Clin Oncol. 2020;38(1):11–19. PMID: 31725351.	**Not related to pMMR/MSS CRC:**included MSI-H/dMMR metastatic CRC.
Andre T, Amonkar M, Norquist JM, et al. *Health-related quality of life in patients with microsatellite instability-high or mismatch repair deficient metastatic colorectal cancer treated with first-line pembrolizumab versus chemotherapy (KEYNOTE-177).* Lancet Oncol. 2021;22(5):665–677. PMID: 33812497.	**Not related to pMMR/MSS CRC:**quality-of-life analysis was confined to MSI-H/dMMR CRC.
Chalabi M, Verschoor YL, Tan PB, et al. *Neoadjuvant Immunotherapy in Locally Advanced Mismatch Repair-Deficient Colon Cancer.* N Engl J Med. 2024;390(21):1949–1958. PMID: 38838311.	**Not related to pMMR/MSS CRC:**study specifically investigated dMMR colon cancer.
Lentz RW, Friedrich TJ, Blatchford PJ, et al. *A Phase II Study of Potentiation of Pembrolizumab with Binimetinib and Bevacizumab in Refractory Microsatellite-Stable Colorectal Cancer.* Clin Cancer Res. 2024;30(17):3768–3778. PMID: 38869830.	**Did not substantively discuss immunotherapy resistance:** primarily assessed treatment efficacy and safety rather than mechanisms or determinants of resistance.
Overman MJ, McCraith S, He B, et al. *Nivolumab plus ipilimumab and cobimetinib in previously treated microsatellite-stable/DNA mismatch repair-proficient metastatic colorectal cancer: the phase II CheckMate 142 trial.*ESMO Gastrointest Oncol. 2026;12:100334. PMID: 42058638.	**Did not substantively discuss immunotherapy resistance:** primarily a clinical activity and safety study without sufficient resistance-focused mechanistic analysis.
Bocobo A, Whitman J, Keenan BP, et al. *Phase II Study of Pembrolizumab Plus Capecitabine and Bevacizumab for Microsatellite Stable/Mismatch Repair-Proficient Metastatic Colorectal Cancer.* Clin Colorectal Cancer. 2026;25(2):271–282. PMID: 41826077.	**Did not substantively discuss immunotherapy resistance:** focused mainly on efficacy and safety of the combination regimen.
Fakih M, Sandhu J, Lim D, et al. *Regorafenib, Ipilimumab, and Nivolumab for Patients With Microsatellite Stable Colorectal Cancer and Disease Progression With Prior Chemotherapy: A Phase 1 Nonrandomized Clinical Trial.* JAMA Oncol. 2023;9(5):627–634. PMID: 36892833.	**Did not substantively discuss immunotherapy resistance:**predominantly evaluated regimen activity and clinical outcomes rather than resistance mechanisms.
Akce M, Farran B, Switchenko JM, et al. *Phase II trial of nivolumab and metformin in patients with treatment-refractory microsatellite stable metastatic colorectal cancer.* J	**Did not substantively discuss immunotherapy resistance:**principally evaluated treatment efficacy and

Immunother Cancer. 2023;11(10):e007235. PMID: 37852737.	safety with limited resistance-specific mechanistic discussion.
Patel MR, Falchook GS, Hamada K, Makris L, Bendell JC. *A phase 2 trial of trifluridine/tipiracil plus nivolumab in patients with heavily pretreated microsatellite-stable metastatic colorectal cancer.* Cancer Med. 2021;10(4):1183–1190. PMID: 33544407.	**Did not substantively discuss immunotherapy resistance:** focused on therapeutic efficacy and safety rather than mechanisms of ICI resistance.
Elez E, Cubillo A, Alfonso PG, et al. *Binimetinib in combination with nivolumab or nivolumab and ipilimumab in patients with previously treated microsatellite-stable metastatic colorectal cancer with RAS mutations in an open-label phase 1b/2 study.* BMC Cancer. 2024;24(1):446. PMID: 38600471.	**Did not substantively discuss immunotherapy resistance:** primarily assessed clinical activity and safety of the treatment combinations.
Gallois C, Emile JF, Kim S, et al. *Pembrolizumab with Capox Bevacizumab in patients with microsatellite stable metastatic colorectal cancer and a high immune infiltrate: The FFCD 1703-POCHI trial.* Dig Liver Dis. 2021;53(10):1254–1259. PMID: 34215534.	**Insufficient methodological or clinical detail:** trial-design/rationale publication without sufficient completed clinical outcome data for the review synthesis.
Passardi A, Sullo FG, Bittoni A, et al. *CombiCoR-Vax trial: study protocol for a phase II, single-arm, multicenter trial of sequential pembrolizumab plus dendritic cell vaccine followed by trifluridine/tipiracil and bevacizumab in refractory microsatellite-stable metastatic colorectal cancer.* BMC Cancer. 2025;25(1):1921. PMID: 41275134.	**Insufficient methodological or clinical detail:** study protocol without completed clinical efficacy results.
Zhang H, Li Y, Xia F, et al. *Study protocol of short-course radiotherapy combined with CAPOX and PD-1 inhibitor for locally advanced colon cancer: a randomised, prospective, multicentre, phase II trial (TORCH-C).* BMJ Open. 2024;14(2):e079442. PMID: 38309748.	**Insufficient methodological or clinical detail:** protocol publication without mature clinical outcome data.

Table S4

**Study**	**Publication type**	**Material or model**	**Main focus**	**Outcome and summary statistics**	**Effect estimates and precision**
**1. Cataldi C, Karaoğlan BB, Liotta E, De Dosso S. Decoding Immunotherapy Response in Colorectal Cancer: Translational Insights Beyond MSI. Cancers (Basel). 2026;18(5):852.**	Review	CRC studies	Immunotherapy response beyond MSI	Reviewed biological and translational determinants of immunotherapy response in CRC. Group summary statistics were not applicable.	Not applicable.
**2. Franke AJ, Skelton WP, Starr JS, et al. Immunotherapy for Colorectal Cancer: A Review of Current and Novel Therapeutic Approaches. J Natl Cancer Inst. 2019;111(11):1131–1141.**	Review	CRC studies	Immunotherapy strategies	Reviewed established and emerging immunotherapeutic approaches in CRC. Group summary statistics were not applicable.	Not applicable.
**3. Yu B, Kang J, Lei H, et al. Immunotherapy for colorectal cancer. Front Immunol.**	Review	CRC studies	Immunotherapy approaches	Reviewed current immunotherapy approaches	Not applicable.

**2024;15:1433315.**				and emerging treatment strategies in CRC. Group summary statistics were not applicable.	
**4. Motta R, Cabezas-Camarero S, Torres-Mattos C, et al. Immunotherapy in microsatellite instability metastatic colorectal cancer: Current status and future perspectives. J Clin Transl Res. 2021;7(4):511–522.**	Review	MSI metastatic CRC	Immunotherapy in MSI CRC	Reviewed clinical evidence and future immunotherapeutic strategies for MSI metastatic CRC. Group summary statistics were not applicable.	Not applicable.
**5. Ros J, Baraibar I, Saoudi N, et al. Immunotherapy for Colorectal Cancer with High Microsatellite Instability: The Ongoing Search for Biomarkers. Cancers (Basel). 2023;15(17):4245.**	Review	MSI-H CRC studies	Predictive biomarkers	Reviewed biomarkers associated with immunotherapy response and resistance in MSI-H CRC. Group summary statistics were not applicable.	Not applicable.
**6. André T, Shiu KK, Kim TW, et al. Pembrolizumab in Microsatellite-Instability-High Advanced Colorectal Cancer. N Engl J Med. 2020;383(23):2207–2218.**	Phase III randomised trial	MSI-H/dMMR metastatic CRC	First-line pembrolizumab	Pembrolizumab prolonged median PFS to **16.5 months versus 8.2 months** with chemotherapy.	**HR 0.60; 95% CI 0.45–0.80.**
**7. Overman MJ, Lonardi S, Wong KYM, et al. Durable Clinical Benefit With Nivolumab Plus Ipilimumab in DNA Mismatch Repair-Deficient/Microsatellite Instability-High Metastatic Colorectal Cancer. J Clin Oncol. 2018;36(8):773–779.**	Phase II trial	dMMR/MSI-H metastatic CRC	Nivolumab plus ipilimumab	Objective response was reported in **55%** of patients; disease control for ≥12 weeks occurred in **80%**.	**ORR 55%; 95% CI 45.2–63.8.**
**8. Lenz HJ, Van Cutsem E, Limon ML, et al. First-Line Nivolumab Plus Low-Dose Ipilimumab for Microsatellite Instability-High/Mismatch Repair-Deficient Metastatic Colorectal Cancer: The Phase II CheckMate 142 Study. J Clin Oncol. 2022;40(2):161–170.**	Phase II trial	Untreated MSI-H/dMMR metastatic CRC	First-line nivolumab plus ipilimumab	Objective response was **69%** and disease control was **84%**.	**ORR 69%; 95% CI 53–82.**
**9. Qu F, Wu S, Yu W. Progress of Immune Checkpoint Inhibitors Therapy for pMMR/MSS Metastatic Colorectal Cancer. Onco Targets Ther. 2024;17:1223–1253.**	Review	pMMR/MSS metastatic CRC studies	ICI therapy in MSS CRC	Reviewed ICI resistance and combination treatment approaches in pMMR/MSS metastatic CRC. Group summary statistics were not applicable.	Not applicable.

**10. Normanno N, Caridi V, Fassan M, et al. Resistance to immune checkpoint inhibitors in colorectal cancer with deficient mismatch repair/microsatellite instability: misdiagnosis, pseudoprogression and/or tumor heterogeneity? Explor Target Antitumor Ther. 2024;5(3):495–507.**	Review	dMMR/MSI CRC studies	ICI resistance	Reviewed misdiagnosis, pseudoprogression and tumour heterogeneity as explanations for apparent or genuine ICI resistance.	Not applicable.
**11. Wang L, Geng H, Liu Y, et al. Hot and cold tumors: Immunological features and the therapeutic strategies. MedComm. 2023;4(5):e343.**	Review	Cancer studies	Hot and cold tumours	Reviewed immunological differences between hot and cold tumours and strategies to improve immune responsiveness.	Not applicable.
**12. Zheng Z, Wieder T, Mauerer B, et al. T Cells in Colorectal Cancer: Unravelling the Function of Different T Cell Subsets in the Tumor Microenvironment. Int J Mol Sci. 2023;24(14):11673.**	Review	CRC studies	T-cell subsets	Reviewed the functions of different T-cell populations within the CRC tumour microenvironment.	Not applicable.
**13. Mezheyeuski A, Micke P, Martín-Bernabé A, et al. The Immune Landscape of Colorectal Cancer. Cancers (Basel). 2021;13(21):5545.**	Review	CRC studies	Immune landscape	Reviewed immune-cell composition and heterogeneity within the CRC microenvironment.	Not applicable.
**14. Pai SI, Cesano A, Marincola FM. The Paradox of Cancer Immune Exclusion: Immune Oncology Next Frontier. Cancer Treat Res. 2020;180:173–195.**	Review	Cancer studies	Immune exclusion	Reviewed mechanisms underlying tumour immune exclusion and their relevance to immunotherapy resistance.	Not applicable.
**15. Liu JL, Yang M, Bai JG, Liu Z, Wang XS. "Cold" colorectal cancer faces a bottleneck in immunotherapy. World J Gastrointest Oncol. 2023;15(2):240–250.**	Review	Cold CRC studies	Immunotherapy resistance	Reviewed biological barriers limiting immunotherapy efficacy in cold CRC and potential strategies to overcome them.	Not applicable.
**16. Li DD, Tang YL, Wang X. Challenges and exploration for immunotherapies targeting cold colorectal cancer. World J Gastrointest Oncol. 2023;15(1):55–68.**	Review	Cold CRC studies	Therapeutic strategies	Reviewed approaches intended to increase immunogenicity and improve immunotherapy response in cold CRC.	Not applicable.
**17. Wu Y, Zhuang J, Qu Z, Yang X, Han S. Advances in immunotyping of colorectal cancer.**	Review	CRC studies	CRC immunotyping	Reviewed immune classifications of CRC and	Not applicable.

**Front Immunol. 2023;14:1259461.**				their potential relevance to prognosis and treatment selection.	
**18. Lin KX, Istl AC, Quan D, et al. PD-1 and PD-L1 inhibitors in cold colorectal cancer: challenges and strategies. Cancer Immunol Immunother. 2023;72(12):3875–3893.**	Review	Cold CRC studies	PD-1/PD-L1 inhibition	Reviewed resistance to PD-1/PD-L1 inhibition and potential combination strategies in cold CRC.	Not applicable.
**19. Hamid MA, Pammer LM, Lentner TK, et al. Immunotherapy for Microsatellite-Stable Metastatic Colorectal Cancer: Can we close the Gap between Potential and Practice? Curr Oncol Rep. 2024;26(10):1258–1270.**	Review	MSS metastatic CRC studies	Immunotherapy in MSS CRC	Reviewed clinical and translational approaches for improving immunotherapy efficacy in MSS metastatic CRC.	Not applicable.
**20. Gilson A, Tan V, Koessler T, et al. Immune Checkpoint Inhibitors for Metastatic Colorectal Cancer: A Systematic Review. Cancers (Basel). 2025;17(13):2125.**	Systematic review	Metastatic CRC studies	ICI efficacy	Synthesised clinical evidence for ICIs in metastatic CRC, with substantially greater activity in MSI than MSS disease.	No single overall effect estimate applicable.
**Study**	Publication type	Material or model	Main focus	Outcome and summary statistics	Effect estimates and precision
**21. Wang R, Lian J, Wang X, et al. Intrinsic resistance and efficacy of immunotherapy in microsatellite instability-high colorectal cancer: A systematic review and meta-analysis. Biomol Biomed. 2023;23(2):198–208.**	Systematic review and meta-analysis	7 clinical trials; 604 MSI-H CRC patients	ICI efficacy and intrinsic resistance	Pooled ORR was 45%, DCR 71%, and progressive disease 25%. (PubMed Central (PMC))	ORR 45% (95% CI 37–52%); DCR 71% (95% CI 62–81%); PD 25% (95% CI 13–37%).(PubMed Central (PMC))
**22. Haag GM, Springfeld C, Grün B, et al. Pembrolizumab and maraviroc in refractory mismatch repair proficient/microsatellite-stable metastatic colorectal cancer – The PICCASSO phase I trial. Eur J Cancer. 2022;167:112–122.**	Phase I trial	20 refractory MMRp/MSS mCRC patients	Pembrolizumab plus maraviroc	Feasibility was 94.7%; ORR 5.3%; median PFS 2.10 months and median OS 9.83 months. (PubMed)	Feasibility 94.7% (90% CI 77.4–99.7%); PFS 2.10 months (95% CI 1.68–2.30); OS 9.83 months (95% CI 5.59–20.02).(PubMed)
**23. Ma SX, Li L, Cai H, Guo TK, Zhang LS. Therapeutic challenge for immunotherapy targeting cold colorectal cancer: A narrative review. World J Clin Oncol. 2023;14(2):81–88.**	Narrative review	Cold CRC studies	Immunotherapy resistance	Reviewed barriers limiting immunotherapy efficacy in cold CRC and potential therapeutic strategies. Group summary statistics were not applicable.	Not applicable.
**24. Guven DC, Kavgaci G, Erul E, et al. The Efficacy of Immune Checkpoint Inhibitors in**	Systematic review	53 clinical trials in	ICI efficacy and combinations	ICI monotherapy or ICI–ICI combinations generally	No single pooled overall effect estimate reported.

**Microsatellite Stable Colorectal Cancer: A Systematic Review. Oncologist. 2024;29(5):e580–e600.**		MSS CRC		produced ORRs <10%; ICI–TKI combinations produced ORRs of approximately 10–40%. (PubMed)	
**25. Wang C, Chevalier D, Saluja J, et al. Regorafenib and Nivolumab or Pembrolizumab Combination and Circulating Tumor DNA Response Assessment in Refractory Microsatellite Stable Colorectal Cancer. Oncologist. 2020;25(8):e1188–e1194.**	Retrospective cohort	18 refractory MSS mCRC patients	Regorafenib plus PD-1 blockade and ctDNA	No objective responses occurred; 5/18 (27.8%) had stable disease and 13/18 (72.2%)progressed. Median PFS was 2 months. (PubMed)	No comparative effect estimate or CI reported.
**26. Cremolini C, Chalabi M, Elez E, et al. Metastatic colorectal cancer: ESMO Clinical Practice Guideline for diagnosis, treatment and follow-up. Ann Oncol. 2026;37(6):759–776.**	Clinical practice guideline	Metastatic CRC evidence	Diagnosis and treatment guidance	Provides evidence-based recommendations for diagnosis, molecular assessment, systemic treatment and follow-up of metastatic CRC. Group summary statistics were not applicable.	Not applicable.
**27. Baraibar I, Mirallas O, Saoudi N, et al. Combined Treatment with Immunotherapy-Based Strategies for MSS Metastatic Colorectal Cancer. Cancers (Basel). 2021;13(24):6311.**	Review	MSS metastatic CRC studies	Immunotherapy combinations	Reviewed clinical evidence for combination approaches intended to overcome immune resistance in MSS mCRC. (PubMed)	Not applicable.
**28. Klempner SJ, Fabrizio D, Bane S, et al. Tumor Mutational Burden as a Predictive Biomarker for Response to Immune Checkpoint Inhibitors: A Review of Current Evidence. Oncologist. 2020;25(1):e147–e159.**	Review	Cancer immunotherapy studies	Tumour mutational burden	Reviewed evidence supporting TMB as a potential predictive biomarker for ICI response. Group summary statistics were not applicable.	Not applicable.
**29. Cornel AM, Mimpen IL, Nierkens S. MHC Class I Downregulation in Cancer: Underlying Mechanisms and Potential Targets for Cancer Immunotherapy. Cancers (Basel). 2020;12(7):1760.**	Review	Cancer studies	MHC-I downregulation	Reviewed mechanisms by which reduced MHC-I expression promotes immune escape and potential approaches for restoring antigen presentation. (PubMed)	Not applicable.
**30. Dhatchinamoorthy K, Colbert JD, Rock KL. Cancer Immune Evasion Through Loss of MHC**	Review	Cancer studies	Antigen-presentation	Reviewed loss of MHC-I antigen presentation as a	Not applicable.

**Class I Antigen Presentation. Front Immunol. 2021;12:636568.**			loss	mechanism of tumour immune escape and resistance to T-cell-based immunotherapy. (PubMed)	
**31. Spranger S, Gajewski TF. A new paradigm for tumor immune escape: β-catenin-driven immune exclusion. J Immunother Cancer. 2015;3:43.**	Perspective/review	Tumour and preclinical studies	β-catenin-mediated immune exclusion	Described tumour-intrinsic β-catenin signalling as a mechanism associated with impaired T-cell infiltration and immune escape. (PubMed)	Not applicable.
**32. Luke JJ, Bao R, Sweis RF, Spranger S, Gajewski TF. WNT/β-catenin Pathway Activation Correlates with Immune Exclusion across Human Cancers. Clin Cancer Res. 2019;25(10):3074–3083.**	Integrative genomic analysis	TCGA; 9,244 tumours across 31 solid tumour types	WNT/β-catenin and immune exclusion	33.9% were non-T-cell-inflamed and 34.2% T-cell-inflamed; β-catenin-pathway mutations were enriched approximately three-fold in non-T-cell-inflamed tumours. (AACR Journals)	Approximately 3-fold enrichment; CI not reported for this comparison.
**33. Yang L, Li A, Lei Q, Zhang Y. Tumor-intrinsic signaling pathways: key roles in the regulation of the immunosuppressive tumor microenvironment. J Hematol Oncol. 2019;12(1):125.**	Review	Cancer studies	Tumour-intrinsic signalling	Reviewed oncogenic signalling pathways that promote an immunosuppressive tumour microenvironment and immune escape. Group summary statistics were not applicable.	Not applicable.
**34. Dąbrowska A, Grubba M, Balihodzic A, et al. The Role of Regulatory T Cells in Cancer Treatment Resistance. Int J Mol Sci. 2023;24(18):14114.**	Review	Cancer studies	Regulatory T cells	Reviewed mechanisms by which Tregs contribute to resistance to immunotherapy, targeted therapy, chemotherapy and radiotherapy. (PubMed)	Not applicable.
**35. Li T, Liu T, Zhu W, et al. Targeting MDSC for Immune-Checkpoint Blockade in Cancer Immunotherapy: Current Progress and New Prospects. Clin Med Insights Oncol. 2021;15:11795549211035540.**	Review	Cancer studies	MDSCs and ICI resistance	Reviewed MDSC-mediated immunosuppression and therapeutic approaches aimed at improving immune-checkpoint blockade. (PubMed)	Not applicable.

**36. Wang S, Wang J, Chen Z, et al. Targeting M2-like tumor-associated macrophages is a potential therapeutic approach to overcome antitumor drug resistance. NPJ Precis Oncol. 2024;8(1):31.**	Review	Cancer studies	M2-like tumour-associated macrophages	Reviewed mechanisms by which M2-like TAMs promote tumour progression and drug resistance and strategies targeting these cells. (PubMed)	Not applicable.
**37. Ceci C, Atzori MG, Lacal PM, Graziani G. Targeting Tumor-Associated Macrophages to Increase the Efficacy of Immune Checkpoint Inhibitors: A Glimpse into Novel Therapeutic Approaches for Metastatic Melanoma. Cancers (Basel). 2020;12(11):3401.**	Review	Metastatic melanoma studies	TAM targeting and ICIs	Reviewed M2-TAM-mediated immune escape and strategies combining TAM targeting with immune-checkpoint inhibition. (PubMed)	Not applicable.
**38. Zhou X, Ni Y, Liang X, Lin Y, An B, He X, Zhao X. Mechanisms of tumor resistance to immune checkpoint blockade and combination strategies to overcome resistance. Front Immunol. 2022;13:915094.**	Review	Cancer studies	ICI resistance	Reviewed tumour-intrinsic and microenvironmental mechanisms of checkpoint-blockade resistance and combination approaches intended to overcome them.	Not applicable.
**39. Hanley CJ, Thomas GJ. T-cell tumour exclusion and immunotherapy resistance: a role for CAF targeting. Br J Cancer. 2020;123(9):1353–1355.**	Perspective/commentary	CAF and tumour studies	CAF-mediated T-cell exclusion	Discussed evidence that CAFs promote T-cell exclusion and immunotherapy resistance and outlined CAF-targeting approaches. (PubMed)	Not applicable.
**40. Maia A, Schöllhorn A, Schuhmacher J, Gouttefangeas C. CAF-immune cell crosstalk and its impact in immunotherapy. Semin Immunopathol. 2023;45(2):203–214.**	Review	Tumour microenvironment studies	CAF–immune-cell crosstalk	Reviewed how CAF interactions with immune cells contribute to treatment failure and immunotherapy resistance. (PubMed)	Not applicable.
**Study**	Publication type	Material or model	Main focus	Outcome and summary statistics	Effect estimates and precision
**41. Salmon H, Franciszkiewicz K, Damotte D, et al. Matrix architecture defines the preferential localization and migration of T cells into the stroma of human lung tumors. J Clin Invest. 2012;122(3):899–910.**	Experimental tissue study	Viable human lung tumour slices	ECM-mediated T-cell exclusion	T cells preferentially accumulated in tumour stroma and migrated poorly through dense matrix; approximately 5-fold more T cellswere present in stroma than tumour islets. (JCI)	No single comparative effect estimate or CI reported.

**42. Nedaeinia R, Najafgholian S, Salehi R, et al. The role of cancer-associated fibroblasts and exosomal miRNAs-mediated intercellular communication in the tumor microenvironment and the biology of carcinogenesis: a systematic review. Cell Death Discov. 2024;10(1):380.**	Systematic review	Cancer studies	CAFs and exosomal miRNAs	Reviewed CAF-derived exosomal miRNAs and their roles in tumour–stromal communication and carcinogenesis. Group summary statistics were not applicable.	Not applicable.
**43. Grout JA, Sirven P, Leader AM, et al. Spatial Positioning and Matrix Programs of Cancer-Associated Fibroblasts Promote T-cell Exclusion in Human Lung Tumors. Cancer Discov. 2022;12(11):2606–2625.**	Translational spatial study	Human lung tumours; scRNA-seq and multiplex imaging	CAF-mediated T-cell exclusion	Identified four major CAF populations; MYH11^{+}αSMA^{+} and FAP^{+}αSMA^{+}CAF populations were associated with dense matrix organisation and T-cell exclusion. (AACR Journals)	No single overall effect estimate applicable.
**44. Fukumura D, Kloepper J, Amoozgar Z, Duda DG, Jain RK. Enhancing cancer immunotherapy using antiangiogenics: opportunities and challenges. Nat Rev Clin Oncol. 2018;15(5):325–340.**	Review	Cancer studies	Antiangiogenic therapy and immunotherapy	Reviewed how vascular normalisation and antiangiogenic treatment may enhance antitumour immunity and improve immunotherapy efficacy. Group summary statistics were not applicable.	Not applicable.
**45. Ribatti D. Immunosuppressive effects of vascular endothelial growth factor. Oncol Lett. 2022;24(4):369.**	Review	Cancer studies	VEGF-mediated immunosuppression	Reviewed mechanisms by which VEGF contributes to an immunosuppressive tumour microenvironment. Group summary statistics were not applicable.	Not applicable.
**46. Hao ZN, Tan XP, Zhang Q, Li J, Xia R, Ma Z. Lactate and Lactylation: Dual Regulators of T-Cell-Mediated Tumor Immunity and Immunotherapy. Biomolecules. 2024;14(12):1646.**	Review	Cancer studies	Lactate, lactylation and T-cell immunity	Reviewed the effects of tumour-derived lactate and lactylation on T-cell function and immunotherapy response. Group summary statistics were not applicable.	Not applicable.
**47. Zhai L, Bell A, Ladomersky E, et al. Immunosuppressive IDO in Cancer: Mechanisms of Action, Animal Models, and Targeting Strategies. Front Immunol.**	Review	Cancer and animal studies	IDO-mediated immunosuppression	Reviewed IDO-mediated immune suppression, experimental models and therapeutic IDO-targeting	Not applicable.

**2020;11:1185.**				strategies. Group summary statistics were not applicable.	
**48. Vito A, El-Sayes N, Mossman K. Hypoxia-Driven Immune Escape in the Tumor Microenvironment. Cells. 2020;9(4):992.**	Review	Cancer studies	Hypoxia and immune escape	Reviewed mechanisms by which tumour hypoxia promotes immune suppression and immune escape. Group summary statistics were not applicable.	Not applicable.
**49. Fu Z, Mowday AM, Smaill JB, Hermans IF, Patterson AV. Tumour Hypoxia-Mediated Immunosuppression: Mechanisms and Therapeutic Approaches to Improve Cancer Immunotherapy. Cells. 2021;10(5):1006.**	Review	Cancer studies	Hypoxia-mediated immunosuppression	Reviewed hypoxia-driven immunosuppressive mechanisms and therapeutic approaches designed to improve immunotherapy.	Not applicable.
**50. Sugiura A, Rathmell JC. Metabolic Barriers to T Cell Function in Tumors. J Immunol. 2018;200(2):400–407.**	Review	Tumour immunology studies	T-cell metabolism	Reviewed metabolic restrictions within tumours that impair T-cell activation and antitumour function.	Not applicable.
**51. Routy B, Le Chatelier E, Derosa L, et al. Gut microbiome influences efficacy of PD-1-based immunotherapy against epithelial tumors. Science. 2018;359(6371):91–97.**	Translational clinical/preclinical study	Patients receiving PD-1/PD-L1 therapy and mouse models	Gut microbiome and ICI efficacy	Antibiotic-associated dysbiosis was associated with reduced ICI benefit; responder-derived FMT improved PD-1 blockade efficacy in mice, with Akkermansia muciniphilaassociated with clinical response. (PubMed)	No single overall effect estimate reported for the combined translational study.
**52. Gopalakrishnan V, Spencer CN, Nezi L, et al. Gut microbiome modulates response to anti-PD-1 immunotherapy in melanoma patients. Science. 2018;359(6371):97–103.**	Prospective translational cohort	112 melanoma patientsreceiving anti-PD-1; faecal analysis in 43	Gut microbiome and anti-PD-1 response	Responders had significantly greater gut microbial diversity and Ruminococcaceae abundance ($P < 0.01$); median PFS was not reached in the high-diversity group versus 232 and 188 days in intermediate- and low-diversity groups. (PubMed)	High vs intermediate diversity: HR 3.60 (95% CI 1.02–12.74); high vs low: HR 3.57 (95% CI 1.02–12.52).(PubMed)
**53. Jiang SS, Xie YL, Xiao XY, et al. Fusobacterium nucleatum-derived succinic acid**	Translational mechanistic	Metastatic CRC	Microbiome-mediated ICI	Nonresponding mCRC patients had greater F.	No single clinical effect estimate or CI reported.

**induces tumor resistance to immunotherapy in colorectal cancer. Cell Host Microbe. 2023;31(5):781–797.e9.**	study	patients and mouse models	resistance	nucleatumabundance and succinic acid; F. nucleatum-derived succinate suppressed cGAS–IFN-β signalling and reduced CD8⁺ T-cell trafficking. (PubMed)	
**54. Shaikh FY, Gills JJ, Sears CL. Impact of the microbiome on checkpoint inhibitor treatment in patients with non-small cell lung cancer and melanoma. EBioMedicine. 2019;48:642–647.**	Review	NSCLC and melanoma studies	Microbiome and ICI treatment	Reviewed associations between intestinal microbiome composition and checkpoint-inhibitor efficacy. Group summary statistics were not applicable.	Not applicable.
**55. von Itzstein MS, Gonugunta AS, Sheffield T, et al. Association between Antibiotic Exposure and Systemic Immune Parameters in Cancer Patients Receiving Checkpoint Inhibitor Therapy. Cancers (Basel). 2022;14(5):1327.**	Observational cohort study	251 ICI-treated cancer patients	Antibiotics and systemic immunity	135/251 (54%)received antibiotics and had poorer response and survival. Antibiotic exposure was also associated with differences in circulating antibodies and cytokines. (PubMed)	No single adjusted effect estimate reported as the principal study result; post-ICI antibiotic exposure was associated with worse radiographic response (P = 0.02). (PubMed Central (PMC))
**56. Crespin A, Le Bescop C, de Gunzburg J, et al. A systematic review and meta-analysis evaluating the impact of antibiotic use on the clinical outcomes of cancer patients treated with immune checkpoint inhibitors. Front Oncol. 2023;13:1075593.**	Systematic review and meta-analysis	123 cohorts; 41,663 patients	Antibiotic exposure and ICI efficacy	Antibiotic use was associated with poorer OS, PFS and treatment response; 11,785 patients (28%) received antibiotics. (PubMed Central (PMC))	OS HR 1.61 (95% CI 1.48–1.76); PFS HR 1.45 (95% CI 1.32–1.60); ORR OR 0.59 (95% CI 0.47–0.76); PD OR 1.86 (95% CI 1.41–2.46).(PubMed Central (PMC))
**57. Sharma P, Hu-Lieskovan S, Wargo JA, Ribas A. Primary, Adaptive, and Acquired Resistance to Cancer Immunotherapy. Cell. 2017;168(4):707–723.**	Review	Cancer studies	Types of immunotherapy resistance	Defined and reviewed mechanisms of primary, adaptive and acquiredresistance to cancer immunotherapy. Group summary statistics were not applicable.	Not applicable.
**58. Barrueto L, Caminero F, Cash L, Makris C, Lamichhane P, Deshmukh RR. Resistance to Checkpoint Inhibition in Cancer**	Review	Cancer studies	Checkpoint inhibitor resistance	Reviewed tumour-intrinsic and extrinsic mechanisms responsible for resistance to	Not applicable.

**Immunotherapy. Transl Oncol. 2020;13(3):100738.**				immune-checkpoint inhibition. Group summary statistics were not applicable.	
**59. Gao J, Shi LZ, Zhao H, et al. Loss of IFN-γ Pathway Genes in Tumor Cells as a Mechanism of Resistance to Anti-CTLA-4 Therapy. Cell. 2016;167(2):397–404.e9.**	Translational mechanistic study	Melanoma patients and mouse models	IFN-γ pathway and CTLA-4 resistance	In the initial cohort, nonresponders had an average of 15.33 IFN-γ-pathway alterations versus 1 in responders; CNAs occurred in 9/12 (75%)nonresponders versus 0/4responders. (PubMed Central (PMC))	CNA difference P = 0.019; no HR or OR reported. (PubMed Central (PMC))
**60. Shin DS, Zaretsky JM, Escuin-Ordinas H, et al. Primary Resistance to PD-1 Blockade Mediated by JAK1/2 Mutations. Cancer Discov. 2017;7(2):188–201.**	Translational mechanistic study	Melanoma, dMMR colon cancer and cell lines	JAK1/2-mediated primary PD-1 resistance	JAK1/2-inactivating mutations occurred in 1/23 melanomaand 1/16 dMMR colon cancer cases treated with PD-1 blockade; both mutation-positive cases failed to respond despite high mutational load. (AACR Journals)	Descriptive frequencies 1/23 and 1/16; no comparative HR/OR or CI reported.
**Study**	Publication type	Material or model	Main focus	Outcome and summary statistics	Effect estimates and precision
**61. Nowicki TS, Hu-Lieskovan S, Ribas A. Mechanisms of Resistance to PD-1 and PD-L1 Blockade. Cancer J. 2018;24(1):47–53.**	Review	Cancer studies	PD-1/PD-L1 resistance	Reviewed tumour-intrinsic and immune-mediated mechanisms of resistance to PD-1/PD-L1 blockade. Group summary statistics were not applicable.	Not applicable.
**62. Zaretsky JM, Garcia-Diaz A, Shin DS, et al. Mutations Associated with Acquired Resistance to PD-1 Blockade in Melanoma. N Engl J Med. 2016;375(9):819–829.**	Translational clinical study	4 metastatic melanoma patients with acquired resistance	Genetic mechanisms of acquired PD-1 resistance	Resistance-associated JAK1 or JAK2 loss-of-function mutations occurred in 2/4 patients, while a B2M truncating mutation occurred in a third. (PubMed)	Descriptive frequencies 2/4 and 1/4; no comparative HR/OR or CI reported. (PubMed)
**63. Zhou B, Gao Y, Zhang P, Chu Q. Acquired Resistance to Immune Checkpoint Blockades: The Underlying Mechanisms and Potential**	Review	Cancer studies	Acquired ICI resistance	Reviewed mechanisms responsible for acquired checkpoint-blockade	Not applicable.

**Strategies. Front Immunol. 2021;12:693609.**				resistance and strategies proposed to overcome them.	
**64. Zhu S, Wang Y, Tang J, Cao M. Radiotherapy induced immunogenic cell death by remodeling tumor immune microenvironment. Front Immunol. 2022;13:1074477.**	Review	Cancer and radiotherapy studies	Radiotherapy-induced ICD	Reviewed how radiotherapy can induce immunogenic cell death and remodel the tumour immune microenvironment to support antitumour immunity.	Not applicable.
**65. Golden EB, Apetoh L. Radiotherapy and immunogenic cell death. Semin Radiat Oncol. 2015;25(1):11–17.**	Review	Cancer studies	Radiotherapy and ICD	Reviewed mechanisms through which radiation-induced tumour-cell death can stimulate antitumour immune responses.	Not applicable.
**66. Liu T, Pei P, Shen W, Hu L, Yang K. Radiation-Induced Immunogenic Cell Death for Cancer Radioimmunotherapy. Small Methods. 2023;7(5):e2201401.**	Review	Cancer studies	Radiation-induced ICD	Reviewed radiation-induced ICD and approaches for exploiting it to enhance cancer radioimmunotherapy.	Not applicable.
**67. Russell L, Peng KW, Russell SJ, Diaz RM. Oncolytic Viruses: Priming Time for Cancer Immunotherapy. BioDrugs. 2019;33(5):485–501.**	Review	Cancer/oncolytic-virus studies	Oncolytic virotherapy	Reviewed how oncolytic viruses promote tumour lysis, antigen release and immune priming to enhance immunotherapy.	Not applicable.
**68. Hemminki O, Dos Santos JM, Hemminki A. Oncolytic viruses for cancer immunotherapy. J Hematol Oncol. 2020;13(1):84.**	Review	Cancer studies	Oncolytic viruses	Reviewed mechanisms, clinical development and combination strategies involving oncolytic viruses in cancer immunotherapy.	Not applicable.
**69. Redwood AJ, Dick IM, Creaney J, Robinson BWS. What's next in cancer immunotherapy? – The promise and challenges of neoantigen vaccination. Oncoimmunology. 2022;11(1):2038403.**	Review	Cancer vaccine studies	Neoantigen vaccination	Reviewed the potential and limitations of personalised neoantigen vaccines for stimulating tumour-specific immunity.	Not applicable.
**70. Barnestein R, Galland L, Kalfeist L, Ghiringhelli F, Ladoire S, Limagne E. Immunosuppressive tumor microenvironment modulation by chemotherapies and targeted therapies to enhance immunotherapy effectiveness. Oncoimmunology.**	Review	Cancer studies	TME modulation	Reviewed how chemotherapy and targeted therapies may modify the immunosuppressive microenvironment and improve immunotherapy	Not applicable.

**2022;11(1):2120676.**				efficacy.	
**71. Zhao Y, Du J, Shen X. Targeting myeloid-derived suppressor cells in tumor immunotherapy: Current, future and beyond. Front Immunol. 2023;14:1157537.**	Review	Cancer studies	MDSC targeting	Reviewed MDSC-mediated immunosuppression and therapeutic approaches aimed at reducing or reprogramming MDSCs.	Not applicable.
**72. Highfill SL, Cui Y, Giles AJ, et al. Disruption of CXCR2-mediated MDSC tumor trafficking enhances anti-PD1 efficacy. Sci Transl Med. 2014;6(237):237ra67.**	Preclinical/translational study	Murine rhabdomyosarcoma; human paediatric sarcoma samples	$CXCR2^{+}$ MDSCs and PD-1 resistance	CXCR2 deficiency or anti-CXCR2 treatment reduced MDSC tumour trafficking and allowed delayed anti-PD-1 therapy to produce significant antitumour effects. Human metastatic paediatric sarcoma also showed elevated CXCR2 ligands. (PubMed)	No single overall effect estimate or CI applicable; experimental comparisons were reported across multiple mouse studies. (PubMed)
**73. Ganesh K, Massagué J. TGF-β Inhibition and Immunotherapy: Checkmate. Immunity. 2018;48(4):626–628.**	Commentary/perspective	Cancer studies	TGF-β and immune exclusion	Discussed evidence linking TGF-β signalling with immune exclusion and the rationale for combining TGF-β inhibition with checkpoint blockade.	Not applicable.
**74. Goel S, Duda DG, Xu L, et al. Normalization of the vasculature for treatment of cancer and other diseases. Physiol Rev. 2011;91(3):1071–1121.**	Review	Cancer and vascular studies	Vascular normalisation	Reviewed the vascular-normalisation concept and how appropriately modulated antiangiogenic therapy may improve tumour perfusion and treatment delivery.	Not applicable.
**75. Hack SP, Zhu AX, Wang Y. Augmenting Anticancer Immunity Through Combined Targeting of Angiogenic and PD-1/PD-L1 Pathways: Challenges and Opportunities. Front Immunol. 2020;11:598877.**	Review	Cancer studies	Antiangiogenic therapy plus ICIs	Reviewed biological and clinical evidence supporting combined inhibition of angiogenic and PD-1/PD-L1 pathways.	Not applicable.
**76. Zhang Y, Brekken RA. Direct and indirect regulation of the tumor immune microenvironment by VEGF. J Leukoc Biol. 2022;111(6):1269–1286.**	Review	Cancer studies	VEGF-mediated immune regulation	Reviewed direct and indirect mechanisms by which VEGF promotes an immunosuppressive tumour microenvironment and affects	Not applicable.

				immune-cell trafficking.	
**77. Schmittnaegel M, Rigamonti N, Kadioglu E, et al. Dual angiopoietin-2 and VEGFA inhibition elicits antitumor immunity that is enhanced by PD-1 checkpoint blockade. Sci Transl Med. 2017;9(385):eaak9670.**	Preclinical experimental study	Genetically engineered and transplant mouse tumour models	ANGPT2/VEGFA inhibition plus PD-1 blockade	Dual ANGPT2/VEGFA blockade produced greater antitumour activity than either single pathway blockade, promoted vascular normalisation and $CD8^+$ T-cell accumulation, and was further enhanced by PD-1 blockade. (PubMed)	No single overall HR/OR or CI reported; effects were assessed across several experimental tumour models. (PubMed)
**78. Leone RD, Powell JD. Fueling the Revolution: Targeting Metabolism to Enhance Immunotherapy. Cancer Immunol Res. 2021;9(3):255–260.**	Review/perspective	Cancer studies	Tumour and immune metabolism	Reviewed metabolic pathways that suppress antitumour immune function and opportunities to target metabolism to enhance immunotherapy.	Not applicable.
**79. Fujiwara Y, Kato S, Nesline MK, et al. Indoleamine 2,3-dioxygenase (IDO) inhibitors and cancer immunotherapy. Cancer Treat Rev. 2022;110:102461.**	Review	Cancer studies and clinical trials	IDO inhibition	Reviewed the biological rationale, clinical evidence and limitations of IDO inhibitors as cancer immunotherapy agents and combination treatments.	Not applicable.
**80. Ricciuti B, Leonardi GC, Puccetti P, et al. Targeting indoleamine-2,3-dioxygenase in cancer: Scientific rationale and clinical evidence. Pharmacol Ther. 2019;196:105–116.**	Review	Preclinical and clinical cancer studies	IDO pathway targeting	Reviewed IDO-mediated immune suppression and clinical evidence for pharmacological IDO inhibition in cancer.	Not applicable.
**Study**	Publication type	Material or model	Main focus	Outcome and summary statistics	Effect estimates and precision
**81. Muller AJ, Manfredi MG, Zakharia Y, Prendergast GC. Inhibiting IDO pathways to treat cancer: lessons from the ECHO-301 trial and beyond. Semin Immunopathol. 2019;41(1):41–48.**	Review	Cancer and clinical-trial evidence	IDO inhibition	Reviewed the failure of ECHO-301 and implications for subsequent development of IDO-targeted cancer therapy.	Not applicable.
**82. Babl N, Decking SM, Voll F, et al. MCT4 blockade increases the efficacy of immune checkpoint blockade. J Immunother Cancer.**	Preclinical/translational study	Human CRC spheroids	MCT4 blockade and ICI efficacy	MCT4 inhibition reduced lactate efflux, increased immune-cell infiltration and	No single overall HR/OR or CI applicable; multiple experimental comparisons

**2023;11(10):e007349.**		and MC38 murine CRC		T-cell activation, delayed tumour growth and improved the activity of PD-L1 blockade. (PubMed)	were reported.
**83. Yu T, Liu Z, Tao Q, et al. Targeting tumor-intrinsic SLC16A3 to enhance anti-PD-1 efficacy via tumor immune microenvironment reprogramming. Cancer Lett. 2024;589:216824.**	Preclinical/translational study	Tumour cell lines, murine models and human datasets	SLC16A3/MCT4 and anti-PD-1 resistance	High SLC16A3 expression was associated with poorer anti-PD-1 activity; genetic or pharmacological inhibition reduced lactate production and improved anti-PD-1 antitumour effects. (PubMed)	No single overall effect estimate or CI reported.
**84. Yang X, Lu Y, Hang J, et al. Lactate-Modulated Immunosuppression of Myeloid-Derived Suppressor Cells Contributes to the Radioresistance of Pancreatic Cancer. Cancer Immunol Res. 2020;8(11):1440–1451.**	Preclinical mechanistic study	Pancreatic cancer models	Lactate, MDSCs and radioresistance	Radiotherapy increased tumour lactate and enhanced MDSC immunosuppression; blocking lactate production or HIF-1α signalling restored T-cell responses and inhibited tumour progression. (PubMed)	No single overall effect estimate or CI reported.
**85. Oh MH, Sun IH, Zhao L, et al. Targeting glutamine metabolism enhances tumor-specific immunity by modulating suppressive myeloid cells. J Clin Invest. 2020;130(7):3865–3884.**	Preclinical mechanistic study	Murine tumour and immune-cell models	Glutamine metabolism and immunosuppression	Glutamine-metabolism inhibition reduced MDSC generation and recruitment, promoted inflammatory macrophages and rendered checkpoint-resistant tumours more responsive to immunotherapy. (PubMed)	No single overall effect estimate or CI reported.
**86. Davar D, Dzutsev AK, McCulloch JA, et al. Fecal microbiota transplant overcomes resistance to anti-PD-1 therapy in melanoma patients. Science. 2021;371(6529):595–602.**	Clinical trial	15 anti-PD-1-refractory melanoma patients	FMT plus pembrolizumab	FMT plus anti-PD-1 provided clinical benefit in 6/15 patients (40%) and was associated with increased $CD8^+$ T-cell activation and reduced IL-8-expressing myeloid cells. (PubMed)	Clinical-benefit rate 40% (6/15); no comparative HR or CI reported.
**87. Baruch EN, Youngster I, Ben-Betzalel G, et al. Fecal microbiota transplant promotes response in immunotherapy-refractory**	Phase I clinical trial	10 anti-PD-1-refractory metastatic	FMT plus anti-PD-1 reinduction	3/10 patients responded, including 2 partial responses and 1 complete response.	ORR 30% (3/10); no CI reported.

**melanoma patients. Science. 2021;371(6529):602–609.**		melanoma patients		(PubMed)	
**88. Zhao W, Lei J, Ke S, et al. Fecal microbiota transplantation plus tislelizumab and fruquintinib in refractory microsatellite stable metastatic colorectal cancer: an open-label, single-arm, phase II trial (RENMIN-215). EClinicalMedicine. 2023;66:102315.**	Phase II single-arm trial	20 refractory MSS/pMMR mCRC patients	FMT + tislelizumab + fruquintinib	Median PFS 9.6 months, median OS 13.7 months, ORR 20% and DCR 95%. (PubMed Central (PMC))	PFS 9.6 months (95% CI 4.1–15.1); OS 13.7 months (95% CI 9.3–17.7); ORR 20% (95% CI 5.7–43.7); DCR 95% (95% CI 75.1–99.9).(PubMed Central (PMC))
**89. Zhao W, Chen Y, Xiao J, et al. Updated outcomes and exploratory analysis of RENMIN-215: tislelizumab plus fruquintinib and fecal microbiota transplantation in refractory microsatellite stable metastatic colorectal cancer. Am J Cancer Res. 2024;14(11):5351–5364.**	Follow-up/exploratory clinical analysis	20 refractory MSS mCRC patients plus real-world comparison cohort	Updated RENMIN-215 outcomes	At 34-month median follow-up, median OS remained 13.7 months and ORR 20%; propensity-score matched analysis showed longer OS with the study regimen. (PubMed)	ORR 20% (95% CI 5.7–43.7); matched OS HR 0.26 (95% CI 0.07–0.95; P=0.042).(PubMed)
**90. Kim Y, Kim G, Kim S, et al. Fecal microbiota transplantation improves anti-PD-1 inhibitor efficacy in unresectable or metastatic solid cancers refractory to anti-PD-1 inhibitor. Cell Host Microbe. 2024;32(8):1380–1393.e9.**	Clinical trial	13 anti-PD-1-refractory advanced solid-cancer patients	FMT plus anti-PD-1	Clinical benefit occurred in 6/13 patients, including 1 partial response and 5 stable diseases; ORR was 7.7% and DCR 46.2%. (PubMed)	ORR 7.7%; DCR 46.2%; no comparative HR or CI reported. (PubMed)
**91. Spencer CN, McQuade JL, Gopalakrishnan V, et al. Dietary fiber and probiotics influence the gut microbiome and melanoma immunotherapy response. Science. 2021;374(6575):1632–1640.**	Translational cohort/preclinical study	Melanoma patients receiving ICI and mouse models	Diet, microbiome and ICI efficacy	Higher dietary fibre was associated with improved PFS in 128 ICI-treated patients, with greatest benefit in patients reporting sufficient fibre intake and no probiotic use. (PubMed)	High vs low dietary fibre: log-rank P=0.047; sufficient fibre/no probiotics vs others: P=0.015. (PubMed)
**92. Hwang SW, Kim MK, Kweon MN. Gut microbiome on immune checkpoint inhibitor therapy and consequent immune-related colitis: a review. Intest Res. 2023;21(4):433–442.**	Review	ICI-treated cancer studies	Gut microbiome and immune-related colitis	Reviewed microbiome associations with ICI efficacy and the development of immune-related colitis.	Not applicable.
**93. Wang B, Yu W, Jiang H, Meng X, Tang D, Liu D. Clinical applications of STING agonists in cancer immunotherapy: current progress and future prospects. Front Immunol. 2024;15:1485546.**	Review	Preclinical and clinical cancer studies	STING agonists	Reviewed clinical development, therapeutic potential and limitations of STING agonists in cancer immunotherapy.	Not applicable.

**94. Wang J, Meng F, Yeo Y. Delivery of STING agonists for cancer immunotherapy. Curr Opin Biotechnol. 2024;87:103105.**	Review	Drug-delivery and cancer studies	STING agonist delivery	Reviewed delivery systems designed to improve tumour targeting and therapeutic activity of STING agonists.	Not applicable.
**95. Hines JB, Kacew AJ, Sweis RF. The Development of STING Agonists and Emerging Results as a Cancer Immunotherapy. Curr Oncol Rep. 2023;25(3):189–199.**	Review	Preclinical and clinical studies	STING agonist development	Reviewed emerging clinical results and challenges associated with STING agonists as cancer immunotherapies.	Not applicable.
**96. Rokade S, Damani AM, Oft M, Emmerich J. IL-2 based cancer immunotherapies: an evolving paradigm. Front Immunol. 2024;15:1433989.**	Review	Cancer immunotherapy studies	IL-2-based therapy	Reviewed conventional and engineered IL-2-based approaches designed to enhance antitumour immunity while reducing toxicity.	Not applicable.
**97. Dong C, Tan D, Sun H, et al. Interleukin-12 Delivery Strategies and Advances in Tumor Immunotherapy. Curr Issues Mol Biol. 2024;46(10):11548–11579.**	Review	Cancer and drug-delivery studies	IL-12 delivery	Reviewed IL-12 delivery platforms and strategies intended to improve local antitumour activity and reduce systemic toxicity.	Not applicable.
**98. Jia Z, Ragoonanan D, Mahadeo KM, et al. IL12 immune therapy clinical trial review: Novel strategies for avoiding CRS-associated cytokines. Front Immunol. 2022;13:952231.**	Clinical-trial review	IL-12 cancer trials	IL-12 efficacy and cytokine toxicity	Reviewed IL-12 clinical trials and approaches intended to retain antitumour activity while reducing cytokine-release-related toxicity.	No single pooled effect estimate applicable.
**99. Fabian KP, Kowalczyk JT, Reynolds ST, Hodge JW. Dying of Stress: Chemotherapy, Radiotherapy, and Small-Molecule Inhibitors in Immunogenic Cell Death and Immunogenic Modulation. Cells. 2022;11(23):3826.**	Review	Cancer and preclinical studies	Treatment-induced ICD	Reviewed how chemotherapy, radiotherapy and small-molecule therapies induce immunogenic cell death or immunogenic modulation capable of enhancing antitumour immunity.	Not applicable.
**100. Khosravi GR, Mostafavi S, Bastan S, et al. Immunologic tumor microenvironment modulators for turning cold tumors hot. Cancer Commun (Lond). 2024;44(5):521–553.**	Review	Cancer studies	Converting cold tumours to hot	Reviewed approaches targeting immune cells, cytokines, vasculature, metabolism and other TME components to improve immune infiltration and	Not applicable.

				immunotherapy response.	
**Study**	Publication type	Material or model	Main focus	Outcome and summary statistics	Effect estimates and precision
**101. Hato T, Zhu AX, Duda DG. Rationally combining anti-VEGF therapy with checkpoint inhibitors in hepatocellular carcinoma. Immunotherapy. 2016;8(3):299–313.**	Review	HCC and tumour-immunity studies	Anti-VEGF plus checkpoint inhibition	Reviewed the rationale for combining VEGF inhibition with immune-checkpoint blockade, including vascular normalisation and reduced immunosuppression. (PubMed)	Not applicable.
**102. Giannone G, Ghisoni E, Genta S, et al. Immuno-Metabolism and Microenvironment in Cancer: Key Players for Immunotherapy. Int J Mol Sci. 2020;21(12):4414.**	Review	Cancer studies	Immunometabolism and TME	Reviewed metabolic and microenvironmental factors influencing antitumour immunity and immunotherapy. Group summary statistics were not applicable.	Not applicable.
**103. Lin A, Jiang A, Huang L, et al. From chaos to order: optimizing fecal microbiota transplantation for enhanced immune checkpoint inhibitors efficacy. Gut Microbes. 2025;17(1):2452277.**	Review	FMT and ICI studies	FMT optimisation	Reviewed approaches for optimising FMT to enhance the efficacy of immune-checkpoint inhibitors. Group summary statistics were not applicable.	Not applicable.
**104. Nerdinger YG, Binder AK, Bremm F, et al. STINGing Cancer: Development, Clinical Application, and Targeted Delivery of STING Agonists. Int J Mol Sci. 2025;26(18):9008.**	Review	Preclinical and clinical cancer studies	STING agonists	Reviewed STING agonist development, clinical application and targeted-delivery strategies.	Not applicable.
**105. Tesniere A, Schlemmer F, Boige V, et al. Immunogenic death of colon cancer cells treated with oxaliplatin. Oncogene. 2010;29(4):482–491.**	Translational mechanistic study	Human/murine CRC cells, mouse models and CRC patients	Oxaliplatin-induced ICD	Oxaliplatin induced calreticulin exposure and HMGB1 release; antitumour effects depended on intact immune signalling, and TLR4 loss-of-function was associated with poorer survival in CRC patients. (PubMed)	No single HR/OR or CI reported in the study summary.
**106. Guan Y, Kraus SG, Quaney MJ, et al.**	Preclinical	Murine	FOLFOX and	FOLFOX reduced $CD8^{+}$ T-	Multiple experimental

**FOLFOX Chemotherapy Ameliorates CD8 T Lymphocyte Exhaustion and Enhances Checkpoint Blockade Efficacy in Colorectal Cancer. Front Oncol. 2020;10:586.**	study	CRC models	CD8⁺ T-cell exhaustion	cell exhaustion, improved effector function and enhanced tumour control when combined with anti-PD-1 therapy. (PubMed)	comparisons; no single overall effect estimate or CI.
**107. Dosset M, Vargas TR, Lagrange A, et al. PD-1/PD-L1 pathway: an adaptive immune resistance mechanism to immunogenic chemotherapy in colorectal cancer. Oncoimmunology. 2018;7(6):e1433981.**	Preclinical/translational study	CRC mouse models and human mCRC samples	FOLFOX-induced adaptive resistance	FOLFOX plus anti-PD-1 produced complete tumour cures in mouse models whereas either treatment alone failed; FOLFOX increased PD-1⁺ CD8⁺ T-cell infiltration and tumour PD-L1 expression. (PubMed)	Experimental group comparisons reported; no single HR/OR or CI applicable.
**108. Lenz HJ, Parikh A, Spigel DR, et al. Modified FOLFOX6 plus bevacizumab with and without nivolumab for first-line treatment of metastatic colorectal cancer: phase 2 results from the CheckMate 9X8 randomized clinical trial. J Immunother Cancer. 2024;12(3):e008409.**	Randomised phase II trial	195 untreated mCRC patients	Nivolumab + mFOLFOX6/bevacizumab	Median PFS was 11.9 months in both groups; ORR was 60% vs 46%with nivolumab plus SOC versus SOC. (BMJ Innovations)	PFS HR 0.81 (95% CI 0.53–1.23; p=0.30).(PubMed Central (PMC))
**109. Antoniotti C, Rossini D, Pietrantonio F, et al. Upfront FOLFOXIRI plus bevacizumab with or without atezolizumab in patients with metastatic colorectal cancer (AtezoTRIBE): a randomised phase 2 trial. Lancet Oncol. 2022;23(7):876–887.**	Randomised phase II trial	218 untreated mCRC patients	Atezolizumab + FOLFOXIRI/bevacizumab	Median PFS was 13.1 months with atezolizumab vs 11.5 months with control. (PubMed)	HR 0.69 (80% CI 0.56–0.85; p=0.012).(PubMed)
**110. Ruan H, Leibowitz BJ, Zhang L, Yu J. Immunogenic cell death in colon cancer prevention and therapy. Mol Carcinog. 2020;59(7):783–793.**	Review	Colon cancer studies	Immunogenic cell death	Reviewed mechanisms and therapeutic implications of ICD in colon cancer. Group summary statistics were not applicable.	Not applicable.
**111. Spiotto M, Fu YX, Weichselbaum RR. The intersection of radiotherapy and immunotherapy: mechanisms and clinical implications. Sci Immunol. 2016;1(3):EAAG1266.**	Review	Cancer studies	Radiotherapy plus immunotherapy	Reviewed mechanisms through which radiation can interact with antitumour immunity and checkpoint-based therapy.	Not applicable.
**112. Lhuillier C, Rudqvist NP, Elemento O,**	Review	Cancer and	Radiation-	Reviewed how radiotherapy	Not applicable.

**Formenti SC, Demaria S. Radiation therapy and anti-tumor immunity: exposing immunogenic mutations to the immune system. Genome Med. 2019;11(1):40.**		radiotherapy studies	induced immunogenicity	may expose tumour antigens and mutations to enhance antitumour immune responses.	
**113. Buchwald ZS, Wynne J, Nasti TH, et al. Radiation, Immune Checkpoint Blockade and the Abscopal Effect: A Critical Review on Timing, Dose and Fractionation. Front Oncol. 2018;8:612.**	Critical review	Radiotherapy–ICI studies	Radiation timing and dose	Reviewed the influence of radiotherapy timing, dose and fractionation on checkpoint blockade and abscopal responses.	Not applicable.
**114. Monjazeb AM, Giobbie-Hurder A, Lako A, et al. A Randomized Trial of Combined PD-L1 and CTLA-4 Inhibition with Targeted Low-Dose or Hypofractionated Radiation for Patients with Metastatic Colorectal Cancer. Clin Cancer Res. 2021;27(9):2470–2480.**	Randomised phase II trial	Metastatic MSS CRC; 18 evaluable patients	Dual ICI plus radiotherapy	Best response was stable disease in 1/18 evaluable patients; median OS was 3.8 months. (AACR Journals)	Median OS 3.8 months (90% CI 2.3–5.7).(AACR Journals)
**115. Parikh AR, Szabolcs A, Allen JN, et al. Radiation therapy enhances immunotherapy response in microsatellite stable colorectal and pancreatic adenocarcinoma in a phase II trial. Nat Cancer. 2021;2(11):1124–1135.**	Phase II single-arm trial	40 MSS CRC and 25 PDAC patients	Radiation + nivolumab/ipilimumab	In CRC, ITT disease control was 25% (10/40); among patients receiving radiation, DCR was 37% (10/27) and ORR approximately 15% (4/27). (PubMed)	CRC DCR 25% (95% CI 13–41%); per-protocol DCR 37% (95% CI 19–58%).(PubMed)
**116. Zhou Y, Liu Z, Yu A, Zhao G, Chen B. Immune Checkpoint Inhibitor Combined with Antiangiogenic Agent Synergistically Improving the Treatment Efficacy for Solid Tumors. Immunotargets Ther. 2024;13:813–829.**	Review	Solid-tumour studies	ICI + antiangiogenic therapy	Reviewed mechanisms and clinical evidence supporting combined checkpoint and antiangiogenic treatment.	Not applicable.
**117. Pei B, Peng S, Chen W, Lai L, Zhou F. Combining Cetuximab and Immunotherapy for Treating MSS/pMMR Colorectal Cancer: Current Evidence and Challenges. Technol Cancer Res Treat. 2025;24:15330338251334209.**	Review	MSS/pMMR CRC studies	Cetuximab plus immunotherapy	Reviewed clinical and mechanistic evidence for combining cetuximab with immunotherapy in MSS/pMMR CRC.	Not applicable.
**118. Martinelli E, Martini G, Famiglietti V, et al. Cetuximab Rechallenge Plus Avelumab in Pretreated Patients With RAS Wild-type Metastatic Colorectal Cancer: The Phase 2 Single-Arm Clinical CAVE Trial. JAMA Oncol. 2021;7(10):1529–1535.**	Phase II single-arm trial	77 pretreated RAS-WT mCRC patients; 71 MSS	Cetuximab + avelumab rechallenge	Median OS was 11.6 months, median PFS 3.6 months, ORR 7.8% and DCR 65%. (JAMA Network)	OS 11.6 months (95% CI 8.4–14.8); PFS 3.6 months (95% CI 3.2–4.1).RAS/BRAF-WT ctDNA was associated with better OS: HR 0.49 (95% CI 0.27–

					0.90).(PubMed)
**119. Yarchoan M, Mohan AA, Dennison L, et al. MEK inhibition suppresses B regulatory cells and augments anti-tumor immunity. PLoS One. 2019;14(10):e0224600.**	Preclinical mechanistic study	CT26 murine CRC and in-vitro models	MEK inhibition and immune modulation	Cobimetinib reduced regulatory B cells and improved T-cell infiltration and anti-PD-1 activity; tumour-draining-node B cells were 28.19% in controls vs 12.9% with MEK inhibition. (PLOS)	28.19% vs 12.9%; p<0.0001 for tumour-draining-node B-cell frequency; no CI reported. (PubMed Central (PMC))
**120. Eng C, Kim TW, Bendell J, et al. Atezolizumab with or without cobimetinib versus regorafenib in previously treated metastatic colorectal cancer (IMblaze370): a phase 3 randomised controlled trial. Lancet Oncol. 2019;20(6):849–861.**	Phase III randomised trial	363 previously treated mCRC patients; predominantly MSS	Atezolizumab ± cobimetinib vs regorafenib	Median OS was 8.87 months with atezolizumab+cobimetinib, 7.10 months with atezolizumab and 8.51 months with regorafenib; the primary endpoint was not met. (PubMed)	Combination vs regorafenib: OS HR 1.00 (95% CI 0.73–1.38; p=0.99).Atezolizumab vs regorafenib: HR 1.19 (95% CI 0.83–1.71; p=0.34).(PubMed)
**Study**	Publication type	Material or model	Main focus	Outcome and summary statistics	Effect estimates and precision
**121. Kim CW, Chon HJ, Kim C. Combination Immunotherapies to Overcome Intrinsic Resistance to Checkpoint Blockade in Microsatellite Stable Colorectal Cancer. Cancers (Basel). 2021;13(19):4906.**	Review	MSS CRC studies	Combination immunotherapy	Reviewed mechanisms of intrinsic ICI resistance in MSS CRC and combination strategies designed to improve response.	Not applicable.
**122. Ros J, Balconi F, Baraibar I, et al. Advances in immune checkpoint inhibitor combination strategies for microsatellite stable colorectal cancer. Front Oncol. 2023;13:1112276.**	Review	MSS CRC studies	ICI combinations	Reviewed emerging ICI-based combination strategies and clinical evidence in MSS CRC.	Not applicable.
**123. Makaremi S, Asadzadeh Z, Hemmat N, et al. Immune Checkpoint Inhibitors in Colorectal Cancer: Challenges and Future Prospects. Biomedicines. 2021;9(9):1075.**	Review	CRC studies	ICI challenges and prospects	Reviewed mechanisms, limitations and future directions of immune-checkpoint inhibition in CRC.	Not applicable.
**124. Mettu NB, Ou FS, Zemla TJ, et al. Assessment of Capecitabine and Bevacizumab With or Without Atezolizumab for the Treatment of Refractory Metastatic Colorectal Cancer: A Randomized Clinical Trial. JAMA**	Randomised phase II trial	128 efficacy-evaluable refractory mCRC	Atezolizumab + capecitabine/bevacizumab	Median PFS was 4.4 months with atezolizumab vs 3.6 months with placebo; in the MSS/pMMR subgroup, benefit was somewhat greater.	Overall PFS HR 0.75 (95% CI 0.52–1.09); MSS/pMMR HR 0.66 (95% CI 0.44–0.99). (PubMed)

**Netw Open. 2022;5(2):e2149040.**		patients		(PubMed)	
**125. Tabernero J, Grothey A, Arnold D, et al. MODUL cohort 2: an adaptable, randomized, signal-seeking trial of fluoropyrimidine plus bevacizumab with or without atezolizumab maintenance therapy for BRAFwt metastatic colorectal cancer. ESMO Open. 2022;7(5):100559.**	Randomised clinical trial	445 BRAF-WT mCRC patients	Atezolizumab maintenance	Adding atezolizumab to fluoropyrimidine/bevacizumab did not significantly improve PFS. (PubMed)	Primary PFS HR 0.92 (95% CI 0.72–1.17; p=0.48); later PFS HR 0.95 (95% CI 0.77–1.18)and OS HR 0.83 (95% CI 0.65–1.05). (PubMed)
**126. Randrian V, Pernot S, Le Malicot K, et al. FFCD 1709-SIRTCI phase II trial: Selective internal radiation therapy plus Xelox, Bevacizumab and Atezolizumab in liver-dominant metastatic colorectal cancer. Dig Liver Dis. 2022;54(7):857–863.**	Phase II trial-design report	Liver-dominant MSS mCRC	SIRT + XELOX + bevacizumab + atezolizumab	Publication describes the prospective single-arm SIRTCI trial and its 9-month PFS primary endpoint; clinical outcome results were not reported in this publication. (PubMed)	Not reported.
**127. Bullock AJ, Schlechter BL, Fakih MG, et al. Botensilimab plus balstilimab in relapsed/refractory microsatellite stable metastatic colorectal cancer: a phase 1 trial. Nat Med. 2024;30(9):2558–2567.**	Phase I trial	148 heavily pretreated MSS mCRC patients; 101 response-evaluable	CTLA-4 + PD-1 blockade	In response-evaluable patients, ORR was 17%, DCR 61%, and median PFS 3.5 months. (PubMed)	ORR 17% (95% CI 10–26%); DCR 61% (95% CI 51–71%); PFS 3.5 months (95% CI 2.7–4.1).(PubMed)
**128. Kim R, Tehfe M, Kavan P, et al. Pembrolizumab Plus mFOLFOX7 or FOLFIRI for Microsatellite Stable/Mismatch Repair-Proficient Metastatic Colorectal Cancer: KEYNOTE-651 Cohorts B and D. Clin Colorectal Cancer. 2024;23(2):118–127.e6.**	Phase Ib trial	31 first-line and 32 previously treated MSS/pMMR mCRC patients	Pembrolizumab + chemotherapy	ORR was 61% with pembrolizumab+mFOLFOX7 and 25% with pembrolizumab+FOLFIRI; efficacy was considered comparable with existing standard therapy. (PubMed)	ORR 61% and 25%, respectively; confidence intervals were not reported in the PubMed abstract. (PubMed)
**129. Li J, Xu X. Immune Checkpoint Inhibitor-Based Combination Therapy for Colorectal Cancer: An Overview. Int J Gen Med. 2023;16:1527–1540.**	Review	CRC studies	ICI combination therapy	Reviewed checkpoint-inhibitor combinations with chemotherapy, targeted therapy, radiotherapy and other immunotherapies.	Not applicable.
**130. Bessudo A, Haseeb AM, Reeves JA, et al. Safety and Efficacy of Vicriviroc (MK-7690) in Combination With Pembrolizumab in Patients**	Randomised phase II trial	40 previously treated	Vicriviroc + pembrolizumab	ORR was only 5% in each vicriviroc dose group, with one partial response in each	ORR 5% (95% CI 0.1–24.9%) in both dose groups. (PubMed)

**With Advanced or Metastatic Microsatellite Stable Colorectal Cancer. Clin Colorectal Cancer. 2024;23(3):285–294.**		MSS/pMMR CRC patients		arm. (PubMed)	
**131. Xu X, Ai L, Hu K, et al. Tislelizumab plus cetuximab and irinotecan in refractory microsatellite stable and RAS wild-type metastatic colorectal cancer: a single-arm phase 2 study. Nat Commun. 2024;15(1):7255.**	Phase II single-arm trial	33 refractory MSS, RAS-WT mCRC patients	Tislelizumab + cetuximab + irinotecan	Confirmed ORR was 33%, DCR 79%, median PFS 7.3 months, and median OS 17.4 months. (PubMed)	PFS 7.3 months (95% CI 5.6–8.6); OS 17.4 months (95% CI 15.9–NR).(PubMed Central (PMC))
**132. Morris VK, Parseghian CM, Bahrambeigi V, et al. Phase 1/2 trial of encorafenib, cetuximab, and nivolumab in microsatellite stable BRAFV600E metastatic colorectal cancer. Cancer Cell. 2025;43(11):2106–2118.e3.**	Phase I/II trial	26 MSS BRAF-V600E mCRC patients	BRAF/EGFR targeting + nivolumab	The triple combination produced an ORR of 50% and median PFS of 7.4 months. (PubMed)	ORR 50% (95% CI 29–71%); PFS 7.4 months (95% CI 5.6–9.6).(PubMed)
**133. Loree JM, Titmuss E, Topham JT, et al. Plasma versus Tissue Tumor Mutational Burden as Biomarkers of Durvalumab plus Tremelimumab Response in Patients with Metastatic Colorectal Cancer in the CO.26 Trial. Clin Cancer Res. 2024;30(15):3189–3199.**	Biomarker analysis of randomised phase II trial	180 refractory mCRC patients	Plasma vs tissue TMB	Tissue TMB ≥10 mutations/Mb did not predict benefit, whereas plasma TMB ≥28 mutations/Mb identified patients with greater benefit from durvalumab+tremelimumab. (PubMed)	Tissue TMB ≥10: HR 0.71 (95% CI 0.28–1.80); plasma TMB ≥28: HR 0.34 (95% CI 0.13–0.85; p=0.022). (PubMed)
**134. Hou W, Yi C, Zhu H. Predictive biomarkers of colon cancer immunotherapy: Present and future. Front Immunol. 2022;13:1032314.**	Review	Colon cancer studies	Predictive biomarkers	Reviewed MSI, PD-L1, TMB, TILs, microbiota, ctDNA and circulating immune cells as potential immunotherapy biomarkers. (PubMed)	Not applicable.
**135. Martin B, Märkl B. Immunologic Biomarkers and Biomarkers for Immunotherapies in Gastrointestinal Cancer. Visc Med. 2019;35(1):3–10.**	Review	Gastrointestinal cancer studies	Immunotherapy biomarkers	Reviewed immune-related biomarkers and their potential prognostic or predictive roles in gastrointestinal cancers.	Not applicable.
**136. Mlecnik B, Lugli A, Bindea G, et al. Multicenter International Study of the Consensus Immunoscore for the Prediction of Relapse and Survival in Early-Stage Colon Cancer. Cancers (Basel). 2023;15(2):418.**	International multicentre prognostic study	1,885 stage I/II colon cancer patients	Consensus Immunoscore	Five-year recurrence-free rates were 78.4%, 88.1% and 93.4% for low, intermediate and high Immunoscore, respectively. (PubMed)	High vs low Immunoscore: HR 0.27 (95% CI 0.18–0.41; p<0.0001); multivariable TTR HR 0.29 (95% CI 0.17–0.50). (PubMed)
**137. Kuang C, Park Y, Augustin RC, et al.**	Phase II	30	Pembrolizumab	Only 1 partial response and 1	ORR 3%; PFS 1.9 months;

**Pembrolizumab plus azacitidine in patients with chemotherapy refractory metastatic colorectal cancer: a single-arm phase 2 trial and correlative biomarker analysis. Clin Epigenetics. 2022;14(1):3.**	single-arm trial	chemotherapy-refractory mCRC patients	+ azacitidine	stable disease were observed; ORR was 3%, median PFS 1.9 months, and median OS 6.3 months. (PubMed)	OS 6.3 months.Confidence intervals were not reported in the abstract. (PubMed)
**138. Bruni D, Angell HK, Galon J. The immune contexture and Immunoscore in cancer prognosis and therapeutic efficacy. Nat Rev Cancer. 2020;20(11):662–680.**	Review	Cancer studies	Immune contexture and Immunoscore	Reviewed the prognostic and therapeutic relevance of tumour immune contexture and the Immunoscore.	Not applicable.
**139. Taube JM, Galon J, Sholl LM, et al. Implications of the tumor immune microenvironment for staging and therapeutics. Mod Pathol. 2018;31(2):214–234.**	Review	Cancer studies	Tumour immune microenvironment	Reviewed pathological assessment of the immune microenvironment and its relevance to tumour staging, prognosis and treatment.	Not applicable.
**140. Angell HK, Bruni D, Barrett JC, Herbst R, Galon J. The Immunoscore: Colon Cancer and Beyond. Clin Cancer Res. 2020;26(2):332–339.**	Review	Colon cancer studies	Immunoscore	Reviewed development and clinical relevance of the Immunoscore as an immune-based prognostic classification in colon cancer and other tumours.	Not applicable.
**Study**	Publication type	Material or model	Main focus	Outcome and summary statistics	Effect estimates and precision
**141. Yu H, Liu Q, Wu K, Tang S. Biomarkers to predict efficacy of immune checkpoint inhibitors in colorectal cancer patients: a systematic review and meta-analysis. Clin Exp Med. 2024;24(1):143.**	Systematic review and meta-analysis	36 studies; 1,867 CRC patients	Predictive biomarkers for ICI efficacy	Lower NLR predicted better prognosis; high TMB predicted ICI response, whereas liver metastasis predicted resistance. PD-L1 was not significantly predictive. (PubMed Central (PMC))	NLR HR 0.37 (95% CI 0.21–0.67); TMB OR 4.83 (95% CI 2.16–10.78); liver metastasis OR 0.32 (95% CI 0.16–0.63); PD-L1 OR 1.01 (95% CI 0.48–2.14).(PubMed Central (PMC))
**142. Kist de Ruijter L, van de Donk PP, Hooiveld-Noeken JS, et al. Whole-body CD8+ T cell visualization before and during cancer immunotherapy: a phase 1/2 trial. Nat Med. 2022;28(12):2601–2610.**	Phase I/II imaging trial	38 evaluable patients with solid tumours receiving ICIs	Whole-body $CD8^+$ T-cell imaging	No tracer-related adverse effects occurred. Median patient tumour SUVmax was 5.2 (IQR 4.0–7.4), with substantial inter- and intrapatient heterogeneity; higher uptake was associated	No single comparative HR/OR with CI was reported as the principal study estimate.

				with longer OS. (PubMed Central (PMC))	
**143. Schwenck J, Sonanini D, Seyfried D, et al. In vivo imaging of CD8+ T cells in metastatic cancer patients: first clinical experience with simultaneous [89Zr]Zr-Df-IAB22M2C PET/MRI. Theranostics. 2023;13(8):2408–2423.**	Prospective clinical imaging study	8 metastatic cancer patients	$CD8^{+}$ T-cell PET/MRI	PET/MRI after 74.2 ± 17.9 MBqtracer administration was well tolerated in all eight patients and enabled visualisation of $CD8^{+}$ T-cell distribution during immunotherapy. (PubMed Central (PMC))	No comparative HR/OR or CI reported.
**144. Farwell MD, Gamache RF, Babazada H, et al. CD8-Targeted PET Imaging of Tumor-Infiltrating T Cells in Patients with Cancer: A Phase I First-in-Humans Study of 89Zr-Df-IAB22M2C. J Nucl Med. 2022;63(5):720–726.**	Phase I first-in-human study	15 patients with metastatic solid tumours	CD8-targeted PET imaging	Tumour tracer uptake occurred in 10/15 (67%)patients, including 7/8 receiving immunotherapy; no drug-related adverse events were observed. (PubMed)	Tumour uptake 67% (10/15); no comparative effect estimate or CI reported.
**145. Dercle L, Sun S, Seban RD, et al. Emerging and Evolving Concepts in Cancer Immunotherapy Imaging. Radiology. 2023;306(1):32–46.**	Review	Cancer imaging studies	Immunotherapy imaging	Reviewed immuno-PET, CT, MRI, FDG-PET, radiomics and imaging approaches for evaluating immunotherapy response and phenomena such as pseudoprogression and hyperprogression. (PubMed)	Not applicable.
**146. Wang JH, Wahid KA, van Dijk LV, et al. Radiomic biomarkers of tumor immune biology and immunotherapy response. Clin Transl Radiat Oncol. 2021;28:97–115.**	Review	Radiomics and immunotherapy studies	Radiomic biomarkers	Reviewed radiomic approaches for characterising tumour immune biology and predicting response to immunotherapy. (PubMed)	Not applicable.
**147. Han X, Cao W, Wu L, Liang C. Radiomics Assessment of the Tumor Immune Microenvironment to Predict Outcomes in Breast Cancer. Front Immunol. 2022;12:773581.**	Retrospective radiomics study	Breast cancer transcriptomic and DCE-MRI datasets	Radiomic ImmunoScore	The immune score independently predicted prognosis; radiomics predicted the immune score with AUCs of 0.899 and 0.815 in training and validation cohorts. (Frontiers)	ImmunoScore: HR 2.42 (95% CI 1.49–3.93; p<0.01).(PubMed)
**148. Saad ED, Coart E, Deltuvaite-Thomas V, et**	Methodologica	Cancer	Clinical-trial	Reviewed methodological	Not applicable.

**al. Trial Design for Cancer Immunotherapy: A Methodological Toolkit. Cancers (Basel). 2023;15(18):4669.**	l review	immunotherapy trials	methodology	issues in immunotherapy trials, including endpoints, delayed effects, non-proportional hazards and trial-design considerations.	
**149. Korn EL, Freidlin B. Adaptive Clinical Trials: Advantages and Disadvantages of Various Adaptive Design Elements. J Natl Cancer Inst. 2017;109(6):djx013.**	Methodological review	Adaptive clinical trials	Adaptive trial design	Reviewed interim monitoring, phase II/III designs, outcome-adaptive randomisation, biomarker-adaptive trials and master protocols. (PubMed)	Not applicable.
**150. Hijazi A, Bifulco C, Baldin P, Galon J. Digital Pathology for Better Clinical Practice. Cancers (Basel). 2024;16(9):1686.**	Review	Digital pathology and cancer studies	Digital pathology and Immunoscore	Reviewed whole-slide imaging, AI-assisted pathology and digital Immunoscore assessment for cancer diagnosis, prognosis and treatment selection. (PubMed)	Not applicable.
**151. Numan H, Tfayli A. Financial barriers to immunotherapy: challenges and potential mitigation strategies: a review paper. Immunotherapy. 2026;18(2):89–97.**	Review	Immunotherapy access and economic studies	Financial barriers	Reviewed high treatment costs, reimbursement limitations, out-of-pocket expenditure, dose optimisation and access barriers, particularly in LMICs. (PubMed)	Not applicable.
**152. Srinivasalu VK, Subramaniam N, Ibrahim M, Das M. Circulating Tumor DNA and Precision Biomarkers in Colorectal Cancer: Implications for Diagnosis, Monitoring, and Management of Advanced Disease. J Gastroenterol Hepatol. 2026;41(7):2030–2042.**	Narrative review	73 human CRC studies	ctDNA and precision biomarkers	Synthesised evidence on ctDNA, genomic, immune, microbiome, radiomic and multiomic biomarkers; ctDNA was identified as the most clinically validated approach for MRD and treatment monitoring. (PubMed)	No single pooled effect estimate applicable.
**153. Zhou Z, Zhou J. Beyond prediction: AI as a mechanistic microscope and digital twin for colorectal cancer immunotherapy. Front Immunol. 2026;17:1746927.**	Review	CRC immunotherapy and multimodal	AI and precision immunotherapy	Reviewed AI-based pathology, imaging and liquid biopsy for response prediction, resistance mapping	Not applicable.

		AI studies		and patient-specific digital-twin approaches. (PubMed)	
**154. Wang Y, Huang P, Li C, Tu S, Yang H. PLGA-based nanoparticles in colorectal cancer immunotherapy: current concepts and future perspectives. Front Pharmacol. 2025;16:1714460.**	Review	CRC nanomedicine studies	PLGA nanoparticles	Reviewed PLGA nanoparticle platforms for immunomodulator delivery, antigen presentation, myeloid reprogramming and combination immunotherapy in CRC. (PubMed)	Not applicable.
**155. Wu J, Wang X, Wang Y, Xun Z, Li S. Application of PLGA in Tumor Immunotherapy. Polymers (Basel). 2024;16(9):1253.**	Review	Tumour nanomedicine studies	PLGA-based immunotherapy	Reviewed PLGA as a carrier for tumour antigens, vaccines, immunoadjuvants and other immunotherapeutic agents. (PubMed)	Not applicable.
**156. Wu Y, Su Y, Wei Y, et al. Nanomedicine for colorectal cancer therapy by remodeling the immune microenvironment. J Control Release. 2026;394:114915.**	Review	Preclinical CRC nanomedicine studies	Immune-microenvironment remodelling	Reviewed nanomedicine strategies designed to remodel the CRC tumour immune microenvironment through targeted drug delivery and multimodal treatment combinations. (PubMed)	Not applicable.
**157. Chen MY, Wang C, Wang YG, Shi M. Adoptive cell therapy in colorectal cancer: Advances in chimeric antigen receptor T cells. World J Gastrointest Oncol. 2025;17(7):106723.**	Review	CRC adoptive-cell-therapy studies	CAR-T and adoptive cell therapy	Reviewed CAR-T, TIL and TCR-engineered therapies in CRC, including antigen heterogeneity, immune suppression and on-target/off-tumour toxicity as major translational barriers. (PubMed Central (PMC))	Not applicable.
**158. Lee NK, Chang JW. Manufacturing Cell and Gene Therapies: Challenges in Clinical Translation. Ann Lab Med. 2024;44(4):314–323.**	Review	Cell and gene therapy manufacturing	Manufacturing and clinical translation	Reviewed manufacturing and quality-control challenges for cell therapies, viral vectors and CAR-T products, including scalability, purification and validation. (PubMed Central (PMC))	Not applicable.
